# The Value of Dysregulated LncRNAs on Clinicopathology and Survival in Non-Small-Cell Lung *Cancer*: A Systematic Review and Meta-Analysis

**DOI:** 10.3389/fgene.2022.821675

**Published:** 2022-04-05

**Authors:** Juan Wang, Xu Han, Ye Yuan, Hao Gu, Xing Liao, Miao Jiang

**Affiliations:** ^1^ Institute of Basic Research in Clinical Medicine, China Academy of Chinese Medical Sciences, Beijing, China; ^2^ The Third Affiliated Hospital of Soochow University, Changzhou, China

**Keywords:** lncRNA, non-small-cell lung cancer, prognosis, clinicopathology, survival

## Abstract

**Background:** There is growing evidence that a number of lncRNAs are involved in the pathogenesis of non-small-cell lung cancer (NSCLC). However, studies on lncRNA expression in NSCLC patients are far from conclusive. Therefore, we performed a systematic review of such studies to collect and examine the evidence on the potential role of lncRNAs in the development of NSCLC.

**Methods:** We systematically searched seven literature databases to identify all published studies that evaluated the expression of one or more lncRNAs in human samples with NSCLC (cases) and without NSCLC (controls) from January 1, 1995 to May 24, 2021. Quality assessment of studies was conducted by using the “Quality in Prognosis Studies” (QUIPS) tool, and the heterogeneity across studies was analyzed with the I-squared statistic and chi-square-based Q-tests. Either fixed or random-effect meta-analysis was performed to summarize effect size to investigate the association between lncRNA expression and overall survival (OS), disease-free survival (DFS), progression-free survival (PFS), and clinicopathological features. The R statistical software program was used to conduct standard meta-analysis.

**Results:** We finally obtained 48 studies with 5,211 patients included in this review after screening. Among the 48 lncRNAs, 38 lncRNAs were consistently upregulated, and 10 were deregulated in patients with NSCLC compared with the control groups. The upregulated lncRNAs were positively associated with histological type: study number (n) = 18, odds ratio (OR) = 0.78, 95% CI: 0.65–0.95 and OR = 1.30, 95% CI: 1.08–1.57, *p* < 0.01; TNM stages: n = 20, OR = 0.41, 95% CI: 0.29–0.57 and OR = 2.44, 95% CI: 1.73–3.44, *p* < 0.01; lymph node metastasis: n = 29, OR = 0.49, 95% CI: 0.34–0.71 and OR = 2.04, 95% CI: 1.40–2.96, *p* < 0.01; differentiation grade: n = 6, OR = 0.61, 95% CI: 0.38–0.99 and OR = 1.63, 95% CI: 1.01–2.64, *p* < 0.01; distant metastasis: n = 9, OR = 0.37, 95% CI: 0.26–0.53 and OR = 2.72, 95% CI: 1.90–3.90, *p* < 0.01; tumor size: n = 16, OR = 0.52, 95% CI: 0.43–0.64 and OR = 1.92, 95% CI: 1.57–2.34, *p* < 0.01; and overall survival [n = 38, hazard ratio (HR) = 1.79, 95% CI = 1.59–2.02, *p* < 0.01]. Especially, five upregulated lncRNAs (linc01234, ZEB1-AS1, linc00152, PVT1, and BANCR) were closely associated with TNM Ⅲa stage (n = 5, OR = 4.07, 95% CI: 2.63–6.28, *p* < 0.01). However, 10 deregulated lncRNAs were not significantly associated with the pathogenesis and overall survival in NSCLC in the meta-analysis (*p* ≥ 0.05).

**Conclusion:** This systematic review suggests that the upregulated lncRNAs could serve as biomarkers for predicting promising prognosis of NSCLC. The prognostic value of downregulated lncRNA in NSCLC needs to be further explored.

**Systematic Review Registration:** (http://www.crd.york.ac.uk/PROSPERO).identifier CRD42021240635.

## Introduction

Lung cancer has been the most common cancer worldwide and leads to approximately 1/5 of all cancer-related deaths (Siegel et al., 2018). There are two main histological types of lung cancer: small-cell lung cancer (SCLC) and non-small-cell lung cancer (NSCLC) (Dela Cruz et al., 2011). NSCLC, one of the most common and invasive type of lung cancer, accounts for more than 85% of lung cancers and can be further divided into three pathological subtypes: adenocarcinoma, squamous cell carcinoma (SCC), and large cell carcinoma (LCC) (Feng et al., 2017). Nearly two-thirds of NSCLC patients have local or distant metastasis at diagnosis with a poor prognosis. The 5-year survival rate of advanced lung cancer is approximately 6% globally (Siegel et al., 2020).

Given that there is still no cure for advanced NSCLC, an effective prognostic factor is essential to get information about disease development, to construct homogeneous groups of patients, and to guide clinical management (Chen R et al., 2020). A prognostic factor is characteristic of a patient or tumor that identifies a better outcome in the absence of treatment, which is defined as a measure of the natural history of the disease (Coate et al., 2009). Both clinical characteristics and molecular-based biomarkers could be used as prognostic factors in NSCLC patients. Established prognostic factors include TNM stages, differentiation grade, tumor size, and lymph node status (Gadgeel and Thakur, 2016). Serological biomarkers, such as carcinoembryonic antigen (CEA), cytokeratin fragment 19 (CYFRA21-1), and neuron-specific enolase (NSE), have been mainly investigated as prognostic markers in NSCLC patients treated with chemotherapy (Ardizzoni et al., 2006; Zhang et al., 2015). Yet superior prognostic factors for NSCLC are still warranted to predict the clinical characteristics and survival of patients.

Long noncoding RNAs (lncRNAs), transcripts of more than 200 nucleotides, generally do not code for proteins, including circular RNAs (circRNAs) and pseudogenes. As they play an essential role in regulating cellular homeostasis and disease progression, such as in cancer (Rinn and Chang, 2012; Peng et al., 2017; Anastasiadou et al., 2018; Slack and Chinnaiyan, 2019), increasing the attention of oncologists that has been attracted on their potential role of being independent prognostic markers for multiple carcinomas in recent years.

The prognostic value of lncRNAs in the pathogenesis and survival of patients with various tumors has been confirmed in a series of basic and clinical studies. A large number of lncRNAs, such as AGAP2-AS1, HOTAIR, MALAT1, MEG3, HOTAIR, CCAT2, H19, etc., are found to be dysregulated in multiple tumors, including breast cancer, hepatocellular carcinoma, kidney cancer, etc. (Gupta et al., 2010; Martens-Uzunova et al., 2014; Li et al., 2016; Wang J et al., 2017; Klingenberg et al., 2017).

However, the diversified results, the heterogeneity of lncRNAs biology and prognosis, as well as the presence of different treatment options make the clinical decision-making process highly varied. Thus, a series of systematic reviews and meta-analyses have been conducted to provide further evidences on the relationship between variants of dysregulated lncRNAs and prognosis of cancer (Chen C et al., 2018; Quan et al., 2018; Tian et al., 2018; Zhang S et al., 2020; Yang et al., 2020; Zhou et al., 2020).

In the year 2016, a meta-analysis that evaluated the expression of lncRNAs and clinical values of patients with NSCLC was published. The relationship between lncRNA levels and overall survival in NSCLC was confirmed, which demonstrated that lncRNAs could be potential prognostic markers for NSCLC. However, the authors indicated that large-scale and comprehensive researches were needed (Wang M et al., 2016). Given that in the past 2 years, the studies of the abnormal expression of lncRNAs have become more extensive and accurate due to technical progress and the rise of targeted therapy, an updated systematic review and meta-analysis would be necessary to illuminate the results timely (Jiang et al., 2019).

In this study, we updated the rereview and aimed to examine the potential role of all the lncRNAs ever investigated in the context of pathogenesis and survival prediction, as well as novel predictors in NSCLC. We applied a field-wide meta-analysis approach to systematically identify and examine all published studies that associated lncRNAs with prognosis in NSCLC, and to quantitatively synthesize data directly related to prognosis (Serghiou et al., 2016).

## Materials and methods

### Protocol and registration

This review has been performed on preferred reporting items for systematic reviews and meta-analysis (PRISMA) (Page et al., 2021). The protocol for the development of this review was prospectively registered on the PROSPERO (International prospective register of systematic reviews) with registration number CRD42021240635 (http://www.crd.york.ac.uk/PROSPERO).

### Search strategy

An online search was conducted in the following databases: PubMed, Web of Science, the Cochrane Library, EBSCO Medline, Chinese Biomedical Literature database (CBM), China National Knowledge Infrastructure (CNKI), and Wanfang database for eligible literature published from January 1, 1995 to November 23, 2020. The search was last updated to include articles published through May 24, 2021. The following keywords and search terms were used: “non-small-cell lung cancer OR lung adenocarcinoma OR lung squamous cell carcinoma OR large cell lung cancer” AND “long-non-coding RNA OR lncRNA.” These items were only the mesh major topics, and specific search terms are displayed in Supplementary Table S1. Additionally, references in relevant articles were also screened to identify potentially eligible literature. There were no restrictions of language.

### Inclusion and exclusion criteria

Studies were included if they met the following criteria: 1) original study on patients diagnosed as NSCLC, 2) those investigated the expression level of lncRNAs in cancer tissue and adjacent tissue or in the corresponding normal control group, 3) those that classified the cases into a high expression group and a low expression group according to the critical value of the lncRNA expression, 4) those that explored the relationship between lncRNA expression and clinicopathological features, 5) those reported the relationship between lncRNA expression and patient survival, and 6) those that provided the odds ratio (OR) or hazard ratio (HR) of survival or sufficient data to calculate them. Studies were excluded if they met any of the following criteria: 1) those that provided insufficient data for pathology, survival data, or curves; 2) those with HRs reported for a combination of lncRNAs; 3) those that included reprocessed data from public databases, such as GEO databases and TCGA databases; and 4) reviews, single case reports, and conference abstracts.

If two or more articles were published by the same author and reported overlapping data, only the one with the most complete data was included. An attempt was made to contact the authors when information was insufficient.

### Data extraction and quality assessment

Data extraction was done independently by two authors (JW and XH) and was supervised by the senior reviewers in case of discrepancies (XH and HG). The following information were extracted from the included studies: 1) basic information including first author’s name, publication year, country of origin; 2) lncRNA information: name, sample size, expression level, detection method, critical values of high expression and low expression; 3) pathological types of non-small-cell lung cancer (e.g., NSCLC, lung adenocarcinoma, or lung squamous cell carcinoma); 4) types of analysis (e.g., univariable or multivariable); 5) clinicopathological features and survival analysis: the number of patients in high and low expression groups, including age, sex, tumor diameter, smoking history, pathological type, TNM stage, lymph node metastasis, distal metastasis, pathological differentiation, pathological stage, and other clinical characteristics; and 6) *p*-values of the correlation between lncRNA expression and clinicopathological features and the original data for calculating the ORs and their 95% CIs, and HRs and their 95% CIs for survival analysis.

The Quality in Prognosis Studies (QUIPS) tool was used to assess the quality of each study by two authors (JW and XH) independently (Hayden et al., 2013).

### Statistical analysis

ORs with 95% CI were used to evaluate the correlation between dysregulated lncRNAs and pathological features of NSCLC. HRs with 95% CI were used to analyze the prognostic value of clinicopathological features in non-small-cell lung cancer. Z test was used to determine the significance of the HR or OR. Q test was used to verify the heterogeneity. If I^2^ > 50%, there was significant heterogeneity between studies, so the random effect model was adopted; otherwise, the fixed effect model was adopted. Subgroup analysis was performed on different groups: upregulated/downregulated lncRNA expression, positive/negative metastasis, early stage (I + II)/advanced stage (III + IV) in TNM (Rami-Porta et al., 2017) staging, poor and high/moderate differentiation, smoking/non-smoking, and LAD/LSCC histological types. At the same time, the subgroup analysis of overall survival was conducted according to the sample size, country, tumor type, and cutoff value. Sensitivity analysis was performed to analyze the sources of heterogeneity by excluding a single study each time. The funnel plot asymmetry test as well as the Begger’s regression test was used to assess publication bias. All statistical analyses were carried out using the meta package of R software. A *p*-value lower than 0.05 was considered statistically significant.

## Results

### Literature search and characteristics of the included studies

**FIGURE 1:**
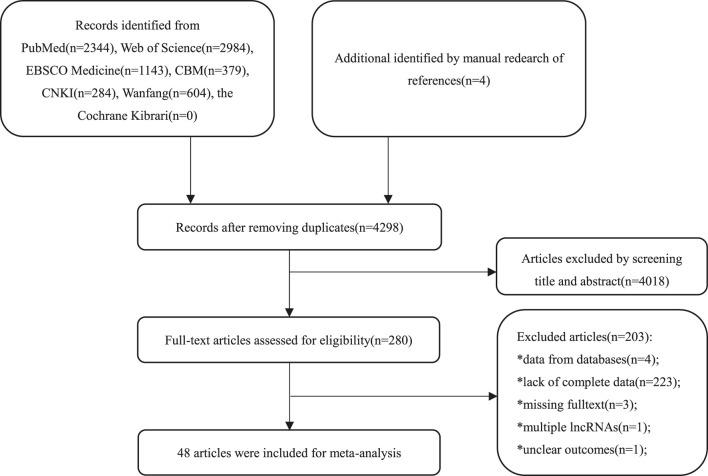
The flowchart of the selection.

A total of 7,742 primary studies from online databases were identified, including PubMed (n = 2,344), Web of Science (n = 2,984), Cochrane Library (n = 0), EBSCO Medline (n = 1,143), CBM (n = 379), CNKI (n = 284), Wanfang (n = 604), and manually retrieved articles (n = 4). In the first step, 3,444 duplicate studies and 4,018 irrelevant studies were excluded after screening the title and abstract. Of the 280 remaining studies, 232 studies were excluded for lack of complete data (n = 223), inclusion of data from public databases (n = 4), missing of full texts (n = 3), analysis of multiple lncRNAs (n = 1), and ambiguous outcomes (n = 1) after the full text had been examined. Finally, 48 studies were included. The flowchart of the selection process is presented in Figure 1.

A total of 48 eligible studies involving 5,211 patients diagnosed with NSCLC were included in this meta-analysis. Of these studies, 38 studies (Hou et al., 2014; Deng et al., 2015; Lin et al., 2015; Wang et al., 2015; Cui et al., 2016; Wan et al., 2016; Xue et al., 2016; Zhang et al., 2016; Li and Jiang, 2017; Liu et al., 2017; Zhang et al., 2017; Song and ZHang, 2018; Tian and Feng, 2018; Xie et al., 2018; Yin et al., 2018; Liu et al., 2019; An et al., 2019; Han et al., 2019; Jin et al., 2019; Navarro et al., 2019; Li et al., 2019; Tang et al., 2019; Wang et al., 2019; Xie et al., 2019; Xu et al., 2019; Yang et al., 2019; Yao et al., 2019; Castellano et al., 2020; Chen J et al., 2020; Chen Z et al., 2020; Fang et al., 2020; Hua et al., 2020; Ma et al., 2020; Wang L et al., 2020; Wang D et al., 2020; Xie and Hu, 2020; Fan et al., 2021; Wang et al., 2021a) with 4,126 patients reported high expression of lncRNAs and 10 (Han et al., 2013; Sun et al., 2014; Xie et al., 2014; Wang Z et al., 2016; Gao et al., 2018; Yu et al., 2019; Zhou et al., 2019; Wang Y et al., 2020; Wang et al., 2021b; Zhang et al., 2021) with 1,085 patients reported low expression of lncRNAs. These studies were published between 2013 and 2020, among which, 46 studies were conducted in China (Hou et al., 2014; Deng et al., 2015; Lin et al., 2015; Wang et al., 2015; Cui et al., 2016; Wan et al., 2016; Xue et al., 2016; Zhang et al., 2016; Li and Jiang, 2017; Liu et al., 2017; Zhang et al., 2017; Song and ZHang, 2018; Tian and Feng, 2018; Xie et al., 2018; Yin et al., 2018; Liu et al., 2019; An et al., 2019; Han et al., 2019; Jin et al., 2019; Li et al., 2019; Tang et al., 2019; Wang et al., 2019; Xie et al., 2019; Xu et al., 2019; Yang et al., 2019; Yao et al., 2019; Han et al., 2013; Sun et al., 2014; Xie et al., 2014; Wang et al., 2016b; Gao et al., 2018; Yu et al., 2019; Zhou et al., 2019; Zhang S et al., 2020; Chen J et al., 2020; Wang Y et al., 2020; Chen Z et al., 2020; Wang D et al., 2020; Fang et al., 2020; Hua et al., 2020; Ma et al., 2020; Xie and Hu, 2020; Wang et al., 2021a; Wang et al., 2021b; Fan et al., 2021; Zhang et al., 2021) and two studies in Spain (Navarro et al., 2019; Castellano et al., 2020). There were 44 studies of NSCLC (Hou et al., 2014; Deng et al., 2015; Lin et al., 2015; Wang et al., 2015; Cui et al., 2016; Wan et al., 2016; Xue et al., 2016; Zhang et al., 2016; Li and Jiang, 2017; Liu et al., 2017; Song and ZHang, 2018; Tian and Feng, 2018; Xie et al., 2018; Yin et al., 2018; Liu et al., 2019; An et al., 2019; Han et al., 2019; Jin et al., 2019; Li et al., 2019; Navarro et al., 2019; Tang et al., 2019; Wang et al., 2019; Xie et al., 2019; Xu et al., 2019; Yang et al., 2019; Yao et al., 2019; Castellano et al., 2020; Wang L et al., 2020; Wang Y et al., 2020; Chen Z et al., 2020; Fang et al., 2020; Hua et al., 2020; Ma et al., 2020; Xie and Hu, 2020; Fan et al., 2021; Han et al., 2013; Sun et al., 2014; Xie et al., 2014; Wang Z et al., 2016; Gao et al., 2018; Yu et al., 2019; Wang D et al., 2020; Wang et al., 2021b; Zhang et al., 2021), three studies of LAD (Zhang et al., 2017; Wang et al., 2021a; Zhou et al., 2019), and one study of LSCC (Chen J et al., 2020). All of the studies applied the quantitative real-time polymerase chain reaction (qRT-PCR) to measure the lncRNA expression level. There were more than 100 samples in 24 studies (Deng et al., 2015; Cui et al., 2016; Wan et al., 2016; Li and Jiang, 2017; Liu et al., 2017; Zhang et al., 2017; Tian and Feng, 2018; Xie et al., 2018; An et al., 2019; Tang et al., 2019; Xie et al., 2019; Xu et al., 2019; Yang et al., 2019; Chen J et al., 2020; Fang et al., 2020; Ma et al., 2020; Xie and Hu, 2020; Sun et al., 2014; Xie et al., 2014; Wang Z et al., 2016; Gao et al., 2018; Wang Y et al., 2020; Wang et al., 2021b; Zhang et al., 2021) and less than 100 samples in 24 studies (Hou et al., 2014; Lin et al., 2015; Wang et al., 2015; Xue et al., 2016; Zhang et al., 2016; Song and ZHang, 2018; Yin et al., 2018; Liu et al., 2019; Han et al., 2019; Jin et al., 2019; Navarro et al., 2019; Li et al., 2019; Wang et al., 2019; Yao et al., 2019; Castellano et al., 2020; Chen Z et al., 2020; Hua et al., 2020; Wang L et al., 2020; Wang D et al., 2020; Fan et al., 2021; Wang et al., 2021a; Han et al., 2013; Yu et al., 2019; Zhou et al., 2019). Overall survival (OS) (Han et al., 2013; Hou et al., 2014; Sun et al., 2014; Xie et al., 2014; Deng et al., 2015; Lin et al., 2015; Wang et al., 2015; Wang Z et al., 2016; Cui et al., 2016; Wan et al., 2016; Xue et al., 2016; Zhang et al., 2016; Li and Jiang, 2017; Liu et al., 2017; Zhang et al., 2017; Gao et al., 2018; Song and ZHang, 2018; Tian and Feng, 2018; Xie et al., 2018; Yin et al., 2018; Liu et al., 2019; An et al., 2019; Han et al., 2019; Jin et al., 2019; Li et al., 2019; Navarro et al., 2019; Tang et al., 2019; Wang et al., 2019; Xie et al., 2019; Xu et al., 2019; Yang et al., 2019; Yao et al., 2019; Yu et al., 2019; Zhou et al., 2019; Wang L et al., 2020; Chen J et al., 2020; Wang Y et al., 2020; Castellano et al., 2020; Chen Z et al., 2020; Wang D et al., 2020; Fang et al., 2020; Hua et al., 2020; Ma et al., 2020; Xie and Hu, 2020; Wang et al., 2021a; Wang et al., 2021b; Fan et al., 2021; Zhang et al., 2021), disease-free survival (DFS) (Wang Z et al., 2016; Cui et al., 2016; Li and Jiang, 2017; Zhang et al., 2017; Gao et al., 2018; Tian and Feng, 2018; Li et al., 2019), progression-free survival (PFS) (Xu et al., 2019; Zhou et al., 2019; Chen J et al., 2020), and recurrence-free survival (RFS) (Li and Jiang, 2017) were investigated to evaluate the outcomes. The univariate and multivariate analyses were used at the same time in 38 studies (Han et al., 2013; Hou et al., 2014; Sun et al., 2014; Xie et al., 2014; Lin et al., 2015; Wang et al., 2015; Wang Z et al., 2016; Cui et al., 2016; Wan et al., 2016; Xue et al., 2016; Zhang et al., 2016; Li and Jiang, 2017; Liu et al., 2017; Zhang et al., 2017; Gao et al., 2018; Tian and Feng, 2018; Xie et al., 2018; Yin et al., 2018; Liu et al., 2019; An et al., 2019; Li et al., 2019; Tang et al., 2019; Wang et al., 2019; Xie et al., 2019; Xu et al., 2019; Yang et al., 2019; Yao et al., 2019; Zhou et al., 2019; Wang L et al., 2020; Chen Z et al., 2020; Wang Y et al., 2020; Fang et al., 2020; Hua et al., 2020; Ma et al., 2020; Xie and Hu, 2020; Wang et al., 2021a; Wang et al., 2021b; Zhang et al., 2021), while multivariate analysis was used in 10 studies (Deng et al., 2015; Song and ZHang, 2018; Han et al., 2019; Jin et al., 2019; Navarro et al., 2019; Yu et al., 2019; Chen J et al., 2020; Wang Y et al., 2020; Castellano et al., 2020; Fan et al., 2021). Cutoff value for high or low lncRNA expression was defined by either mean (Han et al., 2013; Xie et al., 2014; Lin et al., 2015; Xue et al., 2016; Xie et al., 2018; Yang et al., 2019; Wang L et al., 2020; Wang Y et al., 2020), median (Wang Z et al., 2016; Cui et al., 2016; Wan et al., 2016; Zhang et al., 2016; Li and Jiang, 2017; Liu et al., 2017; Zhang et al., 2017; Gao et al., 2018; Song and ZHang, 2018; Tian and Feng, 2018; Yin et al., 2018; Liu et al., 2019; An et al., 2019; Han et al., 2019; Li et al., 2019; Tang et al., 2019; Wang et al., 2019; Xie et al., 2019; Xu et al., 2019; Zhou et al., 2019; Wang D et al., 2020; Chen Z et al., 2020; Fang et al., 2020; Xie and Hu, 2020; Wang et al., 2021a; Wang et al., 2021b; Fan et al., 2021; Zhang et al., 2021) or others (relative expression level, top quartile expression level, the expression level of adjacent normal tissue, the expression level of normal group, and other values through calculation) (Han et al., 2013; Hou et al., 2014; Deng et al., 2015; Wang et al., 2015; Jin et al., 2019; Navarro et al., 2019; Yao et al., 2019; Yu et al., 2019; Chen J et al., 2020; Castellano et al., 2020; Hua et al., 2020; Ma et al., 2020). Five lncRNAs were reported twice and 43 dysregulated lncRNAs were reported in 48 studies. The main characteristics of the included studies are shown in Table 1.

**TABLE 1:** Characteristics of studies included in this meta-analysis.

References no	Author	Year	lncRNAs	Region	Tumor type	Detection method	Expression in tumor	Sample size	Cutoff	Variance analysis	Outcomes	Risk of bias
Wang et al. (2021a)	Wang	2021	FAM83 A-AS1	China	LAD	qRT-PCR	Upregulated	80	Median	UA/MA	OS	Low
Fan et al. (2021)	Fan	2021	SNHG18	China	NSCLC	qRT-PCR	Upregulated	63	Median	MA	OS	Moderate
Wang Y et al. (2020)	Wang	2020	TDRG1	China	NSCLC	qRT-PCR	Upregulated	60	Median	MA	OS	Moderate
Fang et al. (2020)	Fang	2020	XIST	China	NSCLC	qRT-PCR	Upregulated	156	Median	UA/MA	OS	Moderate
Xie and Hu, (2020)	Xie	2020	Linc00691	China	NSCLC	qRT-PCR	Upregulated	177	Median	UA/MA	OS	High
Ma et al. (2020)	Ma	2020	Linc00504	China	NSCLC	qRT-PCR	Upregulated	181	Other	UA/MA	OS	Moderate
Wang L et al. (2020)	Wang	2020	RAB11B-AS1	China	NSCLC	qRT-PCR	Upregulated	97	Mean	UA/MA	OS	High
Hua et al. (2020)	Hua	2020	AC020978	China	NSCLC	qRT-PCR	Upregulated	92	Other	UA/MA	OS	High
Chen Z et al. (2020)	Chen	2020	Linc01234	China	NSCLC	qRT-PCR	Upregulated	45	Median	UA/MA	OS	High
Chen J et al. (2020)	Chen	2020	Linc00173.v1	China	LSCC	qRT-PCR	Upregulated	248	Other	MA	OS, PFS	High
Castellano et al. (2020)	Castellano	2020	p21	Spain	NSCLC	qRT-PCR	Upregulated	56	Other	MA	OS	High
Yao et al. (2019)	Yao	2019	JHDM1D-AS1	China	NSCLC	qRT-PCR	Upregulated	78	Other	UA/MA	OS	Moderate
Yang et al. (2019)	Yang	2019	MNX1-AS1	China	NSCLC	qRT-PCR	Upregulated	154	Mean	UA/MA	OS	Moderate
Xu et al. (2019)	Xu	2019	BLACAT1	China	NSCLC	qRT-PCR	Upregulated	156	Median	UA/MA	OS, PFS	High
Xie et al. (2019)	Xie	2019	Linc01234	China	NSCLC	qRT-PCR	Upregulated	136	Median	UA/MA	OS	Moderate
Wang et al. (2019)	Wang	2019	XIST	China	NSCLC	qRT-PCR	Upregulated	96	Median	UA/MA	OS	High
Tang et al. (2019)	Tang	2019	LBX2-AS1	China	NSCLC	qRT-PCR	Upregulated	165	Median	UA/MA	OS	Moderate
Li et al. (2019)	Li	2019	CACS15	China	NSCLC	qRT-PCR	Upregulated	95	Median	UA/MA	OS, DFS	Moderate
Han et al. (2019)	Han	2019	SNHG16	China	NSCLC	qRT-PCR	Upregulated	66	Median	MA	OS	Moderate
An et al. (2019)	An	2019	Linc00668	China	NSCLC	qRT-PCR	Upregulated	115	Median	UA/MA	OS	High
Am, (2019)	Liu	2019	FAM201 A	China	NSCLC	qRT-PCR	Upregulated	69	Median	UA/MA	OS	Moderate
Navarro et al. (2019)	Navarro	2019	HOTTIP	Spain	NSCLC	qRT-PCR	Upregulated	99	Other	MA	OS	High
Jin et al. (2019)	Jin	2019	ZEB1-AS1	China	NSCLC	qRT-PCR	Upregulated	48	Other	MA	OS	Moderate
Yin et al. (2018)	Yin	2018	AFAP1-AS1	China	NSCLC	qRT-PCR	Upregulated	92	Median	UA/MA	OS	Moderate
Xie et al. (2018)	Xie	2018	ZEB1-AS1	China	NSCLC	qRT-PCR	Upregulated	183	Mean	UA/MA	OS	Moderate
Tian and Feng, (2018)	Tian	2018	Uc.338	China	NSCLC	qRT-PCR	Upregulated	185	Median	UA/MA	OS, DFS	High
Song and ZHang, (2018)	Song	2018	H19	China	NSCLC	qRT-PCR	Upregulated	70	Median	MA	OS	High
Zhang et al. (2017)	Zhang	2017	Linc00152	China	LAD	qRT-PCR	Upregulated	110	Median	UA/MA	OS, DFS	High
Li and Jiang, (2017)	Li	2017	HOXA-AS2	China	NSCLC	qRT-PCR	Upregulated	103	Median	UA/MA	OS,DFS,RFS	Moderate
Liu et al. (2017)	Liu	2017	SUMO1P3	China	NSCLC	qRT-PCR	Upregulated	126	Median	UA/MA	OS	Moderate
Cui et al. (2016)	Cui	2016	PVT1	China	NSCLC	qRT-PCR	Upregulated	108	Median	UA/MA	OS, DFS	High
Xue et al. (2016)	Xue	2016	HOTAIR	China	NSCLC	qRT-PCR	Upregulated	91	Mean	UA/MA	OS	High
Zhang et al. (2016)	Zhang	2016	H19	China	NSCLC	qRT-PCR	Upregulated	70	Median	UA/MA	OS	High
Wan et al. (2016)	Wan	2016	PVT1	China	NSCLC	qRT-PCR	Upregulated	105	Median	UA/MA	OS	High
Wang et al. (2015)	Wang	2015	UCA1	China	NSCLC	qRT-PCR	Upregulated	60	Other	UA/MA	OS	Low
Lin et al. (2015)	Lin	2015	ANRIL	China	NSCLC	qRT-PCR	Upregulated	87	Mean	UA/MA	OS	Moderate
Deng et al. (2015)	Deng	2015	AFAP1-AS1	China	NSCLC	qRT-PCR	Upregulated	121	Other	MA	OS	Moderate
Hou et al. (2014)	Hou	2014	Sox2ot	China	NSCLC	qRT-PCR	Upregulated	83	Other	UA/MA	OS	Moderate
Wang et al. (2021b)	Wang	2021	GAN1	China	NSCLC	qRT-PCR	Downregulated	194	Median	UA/MA	OS	Moderate
Zhang et al. (2021)	Zhang	2021	PINT	China	NSCLC	qRT-PCR	Downregulated	122	Median	UA/MA	OS	Moderate
Wang D et al. (2020)	Wang	2020	NBAT1	China	NSCLC	qRT-PCR	Downregulated	162	Mean	UA/MA	OS	High
Zhou et al. (2019)	Zhou	2019	LOC285194	China	LAD	qRT-PCR	Downregulated	56	Median	UA/MA	OS, PFS	High
Yu et al. (2019)	Yu	2019	Linc00702	China	NSCLC	qRT-PCR	Downregulated	40	Other	MA	OS	Moderate
Gao et al. (2018)	Gao	2018	TCONS00001798	China	NSCLC	qRT-PCR	Downregulated	118	Median	UA/MA	OS, DFS	High
Wang Z et al. (2016)	Wang	2016	TUSC7	China	NSCLC	qRT-PCR	Downregulated	112	Median	UA/MA	OS, DFS	High
Xie et al. (2014)	Xie	2014	HMlincRNA717	China	NSCLC	qRT-PCR	Downregulated	118	Mean	UA/MA	OS	Moderate
Sun et al. (2014)	Sun	2014	BANCR	China	NSCLC	qRT-PCR	Downregulated	113	Other	UA/MA	OS	Moderate
Han et al. (2013)	Han	2013	GAS6-AS1	China	NSCLC	qRT-PCR	Downregulated	50	Mean	UA/MA	OS	Moderate

Note. NSCLC, non-small-cell lung cancer; LAD, lung adenocarcinoma; LSCC, lung squamous cell lung carcinoma; qRT-PCR, quantitative real-time polymerase chain reaction; UA, univariate analysis; MA, multivariate analysis; OS, overall survival; DFS, disease-free survival; PFS, progression-free survival; RFS, recurrence-free survival; lncRNA, long noncoding RNA; other in cutoff: relative expression level, top quartile expression level, the expression level of adjacent normal tissue, the expression level of normal group, and other value through calculation.

After a quality assessment of the literature by QUIPS, 17 studies (Wan et al., 2016; Xue et al., 2016; Zhang et al., 2016; Zhang et al., 2017; Gao et al., 2018; Song and ZHang, 2018; An et al., 2019; Navarro et al., 2019; Xu et al., 2019; Zhou et al., 2019; Wang L et al., 2020; Chen J et al., 2020; Wang D et al., 2020; Wang Y et al., 2020; Xie and Hu, 2020; Wang et al., 2021a; Zhang et al., 2021) were rated as high quality, 27 as medium quality (Han et al., 2013; Hou et al., 2014; Sun et al., 2014; Xie et al., 2014; Deng et al., 2015; Lin et al., 2015; Wang Z et al., 2016; Cui et al., 2016; Li and Jiang, 2017; Liu et al., 2017; Tian and Feng, 2018; Xie et al., 2018; Yin et al., 2018; Liu et al., 2019; Han et al., 2019; Jin et al., 2019; Li et al., 2019; Tang et al., 2019; Wang et al., 2019; Xie et al., 2019; Yang et al., 2019; Yao et al., 2019; Yu et al., 2019; Castellano et al., 2020; Chen Z et al., 2020; Fang et al., 2020; Hua et al., 2020; Ma et al., 2020; Wang et al., 2021b), and 2 as low quality (Wang et al., 2015; Fan et al., 2021). The prognostic factors were measured in a similar way for all participants in the 48 studies. Given that the samples could be collected and preserved at one time, there were no attrition in the 48 studies. Among the 48 studies, 9 studies (Song and ZHang, 2018; An et al., 2019; Xu et al., 2019; Wang L et al., 2020; Chen J et al., 2020; Wang Y et al., 2020; Xie and Hu, 2020; Wang et al., 2021a; Zhang et al., 2021) had a low risk of bias in sample participation, 14 studies (Hou et al., 2014; Deng et al., 2015; Wang Z et al., 2016; Cui et al., 2016; Wan et al., 2016; Xue et al., 2016; Zhang et al., 2016; Liu et al., 2017; Zhang et al., 2017; Gao et al., 2018; Han et al., 2019; Navarro et al., 2019; Xu et al., 2019; Wang Y et al., 2020) had a low risk of bias in outcome measurement, 15 studies (Zhang et al., 2016; Li and Jiang, 2017; Zhang et al., 2017; Gao et al., 2018; Navarro et al., 2019; Wang et al., 2019; Zhou et al., 2019; Wang D et al., 2020; Castellano et al., 2020; Wang Y et al., 2020; Fang et al., 2020; Xie and Hu, 2020; Wang et al., 2021a; Wang et al., 2021b; Zhang et al., 2021) had a low risk of bias in considering important potential confounding factors, and 45 studies (Hou et al., 2014; Deng et al., 2015; Lin et al., 2015; Cui et al., 2016; Wan et al., 2016; Xue et al., 2016; Zhang et al., 2016; Li and Jiang, 2017; Liu et al., 2017; Zhang et al., 2017; Song and ZHang, 2018; Tian and Feng, 2018; Xie et al., 2018; Yin et al., 2018; Liu et al., 2019; An et al., 2019; Han et al., 2019; Jin et al., 2019; Li et al., 2019; Navarro et al., 2019; Tang et al., 2019; Wang et al., 2019; Xie et al., 2019; Xu et al., 2019; Yang et al., 2019; Yao et al., 2019; Wang L et al., 2020; Chen J et al., 2020; Wang D et al., 2020; Castellano et al., 2020; Chen Z et al., 2020; Fang et al., 2020; Hua et al., 2020; Ma et al., 2020; Xie and Hu, 2020; Sun et al., 2014; Xie et al., 2014; Wang Z et al., 2016; Gao et al., 2018; Yu et al., 2019; Zhou et al., 2019; Wang Y et al., 2020; Wang et al., 2021a; Wang et al., 2021b; Zhang et al., 2021) had a low risk of bias in statistical analysis and reporting. The summary of the bias domains, prompting items, and ratings of the QUIPS tool is displayed in Supplementary Table S2. The detailed information of quality assessment for the 48 studies is shown in Supplementary Table S3.

### Association of long noncoding RNA expression with clinicopathological feature of non-small-cell lung cancer

There were 42 studies included in this meta-analysis with clinicopathological features, with 34 involving upregulated lncRNAs and 8 involving downregulated lncRNAs. Table 2 summarizes all the lncRNAs that were related to the clinical feature and pathology of NSCLC.

**TABLE 2:** Summary of lncRNAs related to clinicopathological features of NSCLC.

Clinicopathological feature	Upregulated lncRNAs (carcinogens)	Downregulated lncRNAs (antioncogenes)
Age	FAM83A-AS1, XIST, TDRG1, linc00691, linc00504, FAB11B-AS1, AC020978, linc01234, linc00173.v1, JHDM1D-AS1, MNX1-AS1, BLACAT1, LBX2-AS1, CACS15, SNHG16, linc00668, FAM201A, AFAP1-AS1, ZEB1-AS1, uc.338, H19, linc00152, HOXA-AS2, SUMO1P3, PVT1, UCA1, ANRIL.	NBAT1, LOC285194, TCONS00001798, TUSC7, HMlincRNA717, BANCR, GAS6-AS1
Gender	FAM83A-AS1, XIST, TDRG1, linc00691, linc00504, FAB11B-AS1, AC020978, linc01234, linc00173.v1, JHDM1D-AS1, MNX1-AS1, BLACAT1, LBX2-AS1, CACS15, SNHG16, linc00668, FAM201A, AFAP1-AS1, ZEB1-AS1, uc.338, H19, linc00152, HOXA-AS2, SUMO1P3, PVT1, UCA1, ANRIL.	PINT, NBAT1, LOC285194, TCONS00001798, TUSC7, HMlincRNA717, BANCR, GAS6-AS1
Tumor size	FAM83A-AS1, XIST, TDRG1, linc00691, linc00504, FAB11B-AS1, linc01234, MNX1-AS1, LBX2-AS1, CACS15, SNHG16, linc00668, AFAP1-AS1, ZEB1-AS1, uc.338, H19, HOXA-AS2, SUMO1P3, PVT1, UCA1, ANRIL.	PINT, NBAT1, LOC285194, TCONS00001798, TUSC7, HMlincRNA717, BANCR, GAS6-AS1
Smoking	XIST, linc00691, MNX1-AS1, BLACAT1, LBX2-AS1, CACS15, SNHG16, linc00668, HOXA-AS2, PVT1, FAB11B-AS1, H19, SUM01P3, linc01234, ZEB1-AS1	PINT, NBAT1, TCONS00001798, BANCR, GAS6-AS1
Histological type	Linc00691, linc00504, BLACAT1, linc01234, FAM201A, ZEB1-AS1, VPS9D1-AS1, HOXA-AS2, ANRIL, HOTAIR, FAB11B-AS1, H19, SUM01P3, PVT1	NBAT1, LOC285194, TCONS00001798, TUSC7, HMlincRNA717, BANCR, GAS6-AS1
Histological differentiation	FAM83A-AS1, linc00691, MNX1-AS1, linc01234, LBX2-AS1, SNHG16, linc00668, ZEB1-AS1, HOXA-AS2, PVT1, UCA1, FAB11B-AS1, H19, SUM01P3, AC020978	PINT, NBAT1, LOC285194, HMlincRNA717, GAS6-AS1
TNM stage	FAM83A-AS1, XIST, TDRG1, linc00691, linc00504, JHDM1D-AS1, BLACAT1, linc01234, LBX2-AS1, CACS15, SNHG16, linc00668, FAM201A, 1, ZEB1-AS1, uc.338, linc00152, HOXA-AS2, PVT1, ANRIL, H19, SUM01P3, AC020978	PINT, NBAT1, LOC285194, TUSC7, BANCR.
Lymph node metastasis	FAM83A-AS1, SNHG18, XIST, TDRG1, linc00691, linc00504, JHDM1D-AS1, MNX1-AS1, linc01234, LBX2-AS1, SNHG16, linc00668, uc.338, HOXA-AS2, UCA1, ANRIL, FAB11B-AS1, H19, SUM01P3, AC020978, ZEB1-AS1, PVT1, AFAP-AS1	PINT, NBAT1, LOC285194, TCONS00001798, HMlincRNA717, BANC
Distant metastasis	Linc00504, JHDM1D-AS1, XIST, uc.338, HOXA-AS2, PVT1, FAB11B-AS1, AC020978, AFAP-AS1	—

Of all the 48 lncRNAs, 6 lncRNAs (p21, HOTTIP, HOTAIR, Sox2ot, GAN1, and linc00702) were not provided with detailed data about their relationship with clinicopathological features of NSCLC. The expression of the remaining 42 lncRNAs (linc00691, XIST, PVT1, TUSC7, BANCR, etc.) was related to one or more clinical parameters of NSCLC, including age, gender, tumor size, lymph node metastasis, histological type, TNM stage, differentiation grade, smoking, and distant metastasis (*p* < 0.05).

We observed that an upregulated lncRNA expression level was related to age [studies number (n) = 20, odds ratio (OR) = 1.00, 95% CI: 0.85–1.19 (age ≤ 60) and OR = 1.00, 95% CI: 0.84–1.18 (age > 60), *p* = 0.97], gender [n = 33, OR = 1.04, 95% CI: 0.91–1.19 (male) and OR = 0.96, 95% CI: 0.84–1.10 (female), *p* = 0.58], smoking [n = 16, OR = 0.99, 95% CI: 0.82–1.21, *p* = 0.94 (never) and OR = 1.04, 95% CI: 0.85–1.26 (ever), *p* = 0.72], histological type [n = 18, OR = 0.78, 95% CI: 0.65–0.95 (LSCC) and OR = 1.30, 95% CI: 1.08–1.57 (LAD), *p* < 0.01], TNM stage [n = 20, OR = 0.41, 95% CI: 0.29–0.57 (TNM Ⅰ/Ⅱ stages) and OR = 2.44, 95% CI: 1.73–3.44 (TNM Ⅲ/Ⅳ stages), *p* < 0.01], lymph node metastasis [n = 29, OR = 0.49, 95% CI: 0.34–0.71 (negative) and OR = 2.04, 95% CI: 1.40–2.96 (positive), *p* < 0.01], differentiation grade [n = 6, OR = 0.61, 95% CI: 0.38–0.99 (high/moderate) and OR = 1.63, 95% CI: 1.01–2.64 (poor), *p* < 0.01], distant metastasis [n = 9, OR = 0.37, 95% CI: 0.26–0.53 (negative) and OR = 2.72, 95% CI: 1.90–3.90 (positive), *p* < 0.01], and tumor size [n = 16, OR = 0.52, 95% CI: 0.43–0.64 (tumor size ≤ 3 cm) and OR = 1.92, 95% CI: 1.57–2.34 (tumor size > 3 cm), *p* < 0.01].

However, the downregulated lncRNA level was not significantly associated with clinicopathological features. The results showed that downregulated lncRNA expression level was related to age [n = 6, OR = 1.08, 95% CI = 0.77–1.52 (age ≤ 60), and OR = 0.93, 95% CI = 0.66–1.30 (age > 60), *p* = 0.49], gender [n = 8, OR = 1.04, 95% CI: 0.79–0.37 (male) and OR = 0.96, 95% CI: 0.73–1.27 (female), *p* = 0.70], smoking [n = 6, OR = 1.10, 95% CI: 0.80–1.53 and OR = 0.91, 95% CI: 0.80–1.53 (ever), *p* = 0.55], histological type [n = 7, OR = 1.10, 95% CI: 0.81–1.50 (LSCC) and OR = 0.91, 95% CI: 0.67–1.24 (LAD), *p* = 0.55], TNM stage [n = 3, OR = 1.15, 95% CI: 0.27–4.96 (TNM Ⅰ/Ⅱ stages) and OR = 0.87, 95% CI: 0.20–3.74 (TNM Ⅲ/Ⅳ stages), *p* = 0.85], lymph node metastasis [n = 6, OR = 1.09, 95% CI: 0.46–2.58 (negative) and OR = 0.91, 95% CI: 0.39–2.16 (positive), *p* = 0.84], and tumor size [n = 6, OR = 1.06, 95% CI: 0.55–2.05 (tumor size ≤ 3 cm) and OR = 0.94, 95% CI: 0.49–1.83 (tumor size > 3 cm), *p* = 0.87].

**FIGURE 2:**
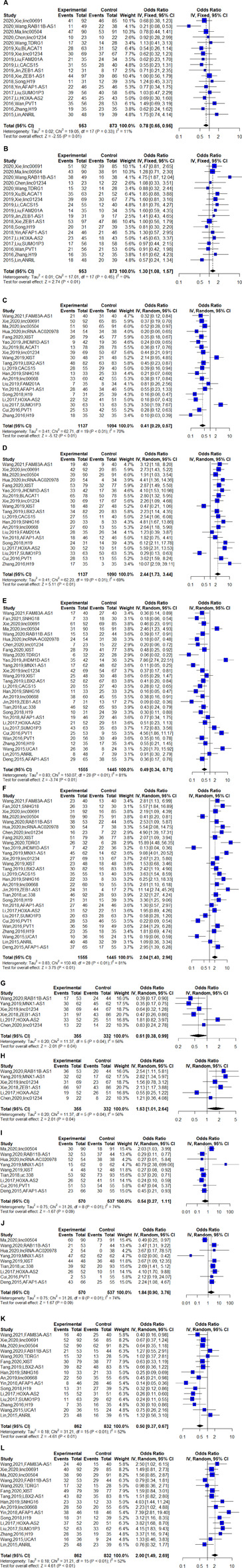
Forest plots of the associations between the expression of long noncding RNAs (lncRNAs) and clinical features of non-small-cell lung cancer (NSCLC) [There were six clinical features that were significantly associated with the upregulated lncRNAs in NSCLC, **(A)** the histological type of lung squamous cell carcinoma (LSCC); **(B)** the histological type of lung adenocarcinoma (LAD); **(C)** the Ⅰ/Ⅱ TNM stages; **(D)** the Ⅲ/Ⅳ TNM stages; **(E)** negative lymph node metastasis; **(F)** positive lymph node metastasis; **(G)** high or moderate differentiation grade; **(H)** poor differentiation grade; **(I)** negative distant metastasis; **(J)** positive distant metastasis; **(K)** tumor size no larger than 3 cm; and **(L)** tumor size larger than 3 cm].

The pooled ORs and 95% confidence intervals (CIs) of the clinicopathological characteristics for all the enrolled studies are presented in Supplementary Table S4, and the significant associations are shown in Figure 2.

**FIGURE 3:**
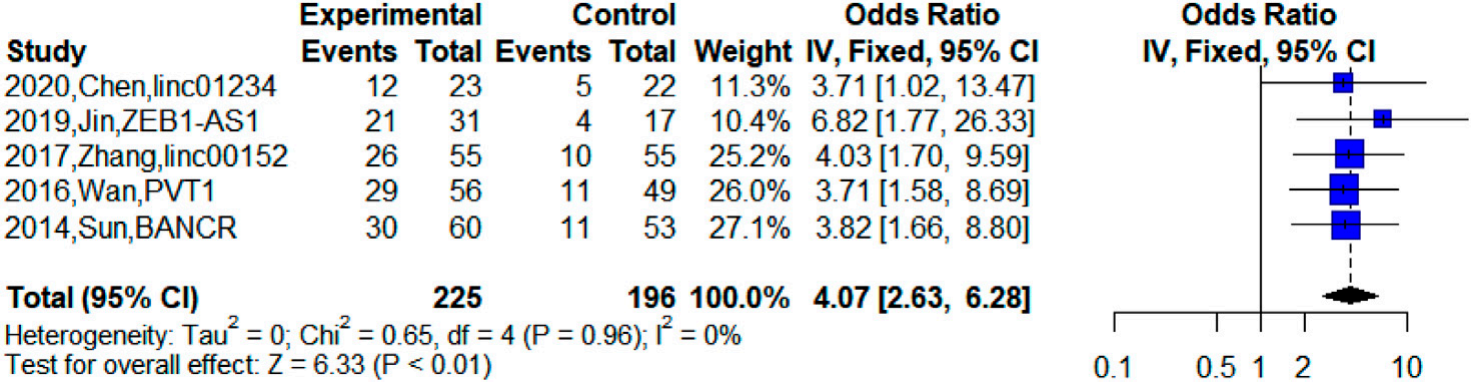
Forest plots of the relationship between TNM Ⅲa stage and the lncRNA expression level.

Besides, five studies reported upregulated lncRNA expression in patients at TNM Ⅲa stage (Sun et al., 2014; Wan et al., 2016; Zhang et al., 2017; Jin et al., 2019; Chen Z et al., 2020). The meta-analysis indicated that high expression of lncRNAs was significantly related to TNM Ⅲa stage (n = 5, OR = 4.07, 95% CI: 2.63–6.28, *p* < 0.01). This suggested that the abnormal expression of lncRNAs was significantly associated with TNM Ⅲa stage and the upregulated lncRNAs may indicate poor prognosis for NSCLC patients at the TNM Ⅲa stage. However, we could not explore the difference between TNM Ⅲa stage and TNM Ⅲb stage due to insufficient data, which was difficult in the current clinical treatment of NSCLC. Figure 3 shows the relationship between TNM Ⅲa stage and upregulated lncRNAs.

### Prognostic value of long noncoding RNA expression for non-small-cell lung cancer survival

**FIGURE 4:**
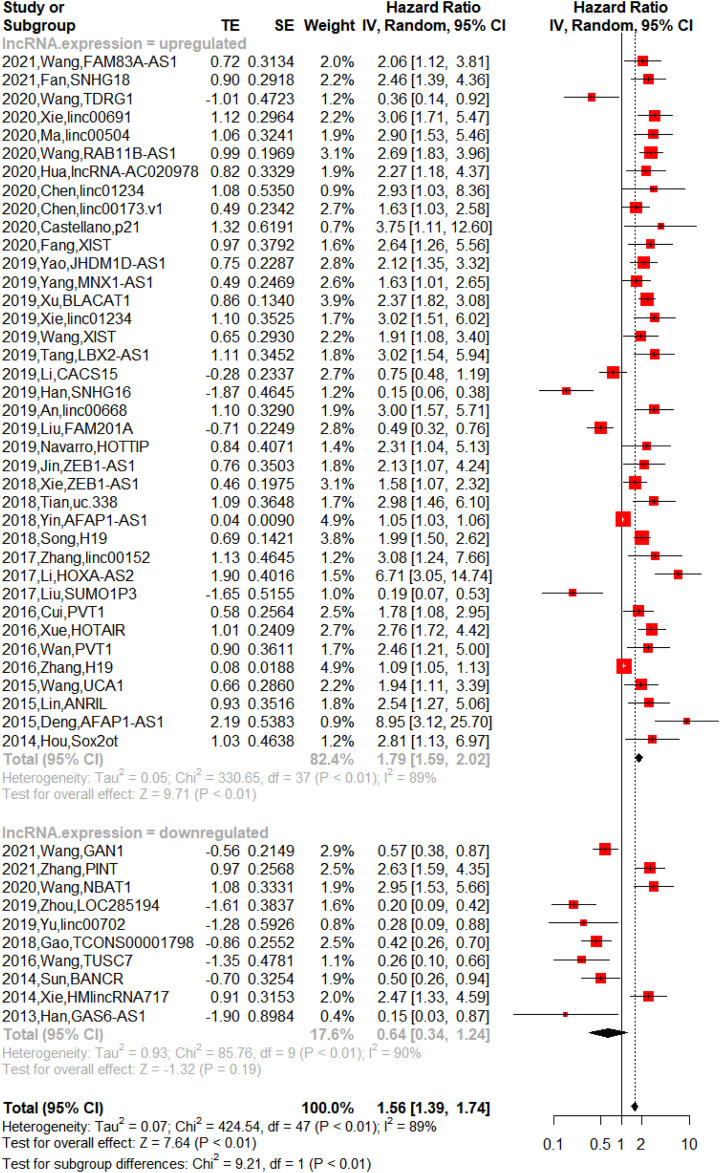
Forest plots of hazard ratio (HR) for association between lncRNA expression and overall survival [There was heterogeneity between the upregulated lncRNA group and downregulated lncRNA group (*p* < 0.01). The upregulated lncRNAs indicated shorter overall survival (OS) in NSCLC (*p* < 0.01), and downregulated lncRNAs showed no significant association with OS in NSCLC (*p* = 0.19)].

The upregulated lncRNAs were identified as carcinogens, while the downregulated lncRNAs were antioncogenes. The 48 lncRNAs were divided into two groups (upregulated lncRNA group and downregulated lncRNA group) in this meta-analysis. Among them, patients with high expression of lncRNAs had shorter overall survival (OS) [n = 38, hazard ratio (HR) = 1.79, 95% CI = 1.59–2.02, *p* < 0.01), while the downregulated lncRNAs were not significantly associated with poor OS (n = 10, HR = 0.64, 95% CI:0.3–1.24, *p* = 0.19). As shown in Figure 4, 48 lncRNAs in 5,211 patients were all included in the meta-analysis for overall survival of NSCLC, and the heterogeneity was high (upregulated lncRNA group: *p* < 0.01, I^2^ = 89%, downregulated lncRNA group: *p* < 0.01, I^2^ = 90%). The influence of a single study on the overall meta-analysis was investigated by omitting the study one at a time, and the omission of any study in the upregulated lncRNA group made no significant difference (Supplementary Table S5); however, we found that the *p*-value was lower than 0.0001 if three studies were omitted (2021-Zhang-PINT, 2020-Wang-NBAT1, and 2014-Xie-HMlincRNA717) in the downregulated lncRNA group (Supplementary Table S6). We analyzed the three studies, but no element indicated that they were distinct. Therefore, we performed subgroup analysis based on country, histological type, sample size, and cutoff value, and similarly, heterogeneity was also assessed in a stratified analysis, where no significant changes in heterogeneity after subgrouping was proven (Table 3).

**TABLE 3:** Subgroup meta-analysis of pooled hazard ratio (HR) for overall survival.

Subgroups	Studies(n)	HR	95% CI	*p*-Value	I^2^	Model	Differences between groups (*p*-value)
Sample size							
≤100	24	1.36	(1.1913, 1.5475)	<0.01	88%	Random	0.09
>100	24	1.82	(1.3364, 2.4746)	<0.01	85%	Random	
Country							
China	46	1.54	(1.3708, 1.7233)	<0.01	89%	Random	0.11
Spain	2	2.67	(1.3722, 5.2060)	<0.01	0%	Random	
Histological type							
LAD	3	1.08	(0.2077, 5.5698)	<0.01	93%	Random	0.89
NSCLC	44	1.58	(1.4101, 1.7785)	<0.01	89%	Random	
LSCC	1	1.63	(1.0299, 2.5797)	0.04	—	—	—
Cutoff value							
Median	28	1.33	(1.1692, 1.5216)	<0.01	90%	Random	0.01
Mean	8	2.11	(1.5685, 2.8502)	<0.01	57%	Random	
Other	12	1.92	(1.3015, 2.8209)	<0.01	73%	Random	

**FIGURE 5:**
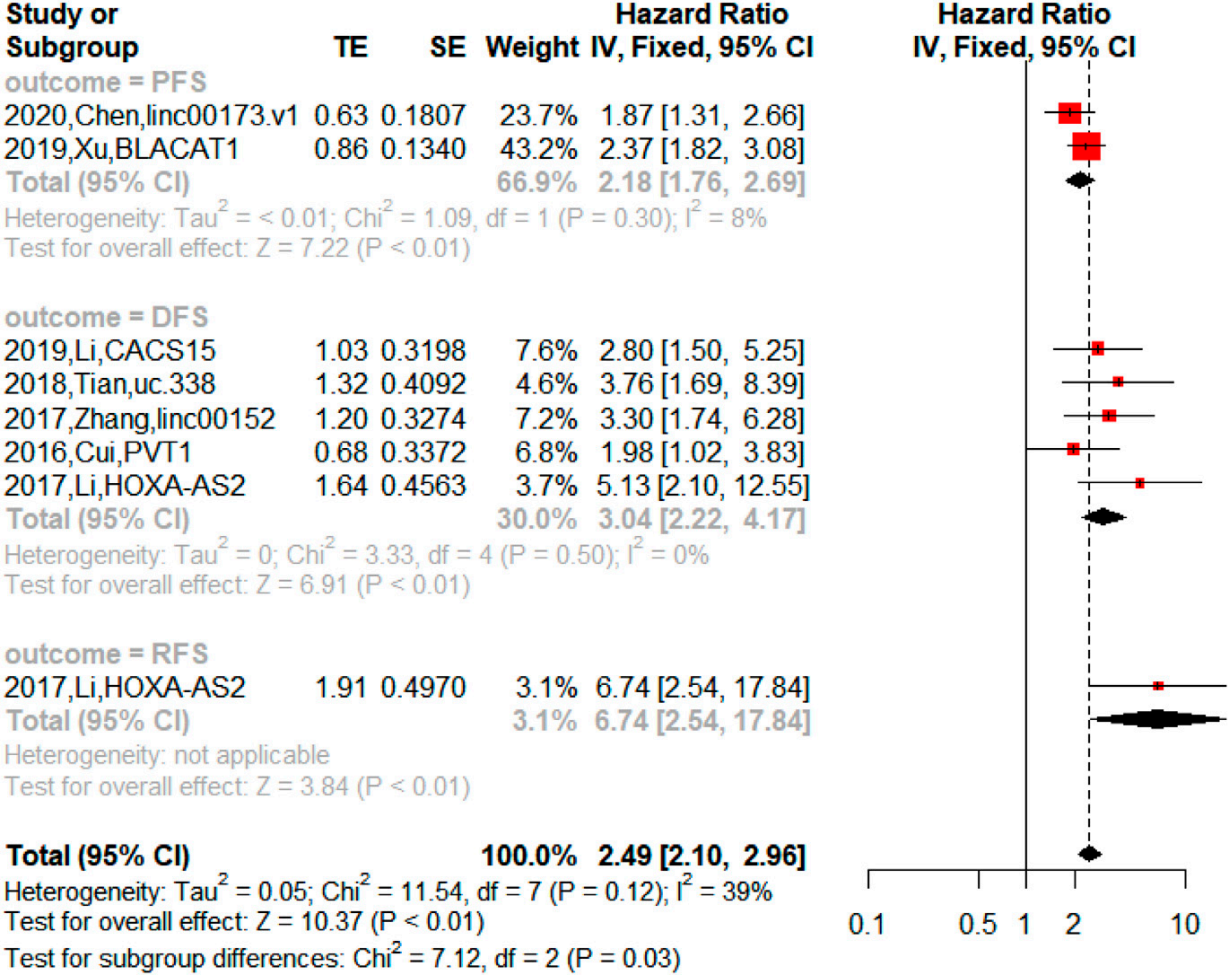
Forest plots of the association between upregulated lncRNAs and progression-free survival (PFS), disease-free survival (DFS), and recurrence-free survival (RFS) in NSCLC.

**FIGURE 6:**
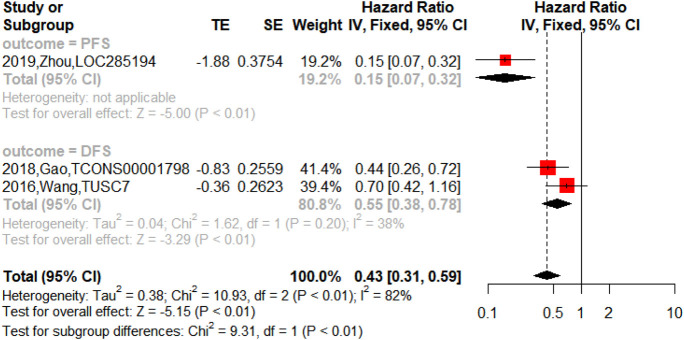
Forest plots of the association between downregulated lncRNAs and PFS and DFS in NSCLC.

On the other hand, the upregulated lncRNAs were also associated with PFS (n = 2, HR = 2.18, 95% CI = 1.76–2.69, *p* < 0.01), DFS (n = 5, HR = 3.04, 95% CI = 2.22–4.17, *p* < 0.01) and RFS (n = 1, HR = 6.74, 95% CI = 2.54–17.84, *p* < 0.01) in NSCLC as shown in Figure 5. The downregulated lncRNAs were found to exert an impact on PFS (n = 1, HR = 0.15, 95% CI = 0.07–0.32, *p* < 0.01) and DFS (n = 2, HR = 0.55, 95% CI = 0.38–0.78, *p* < 0.01) in NSCLC as shown in Figure 6.

## The mechanism of upregulated long noncoding RNAs as prognostic biomarkers in non-small-cell lung cancer

**FIGURE 7:**
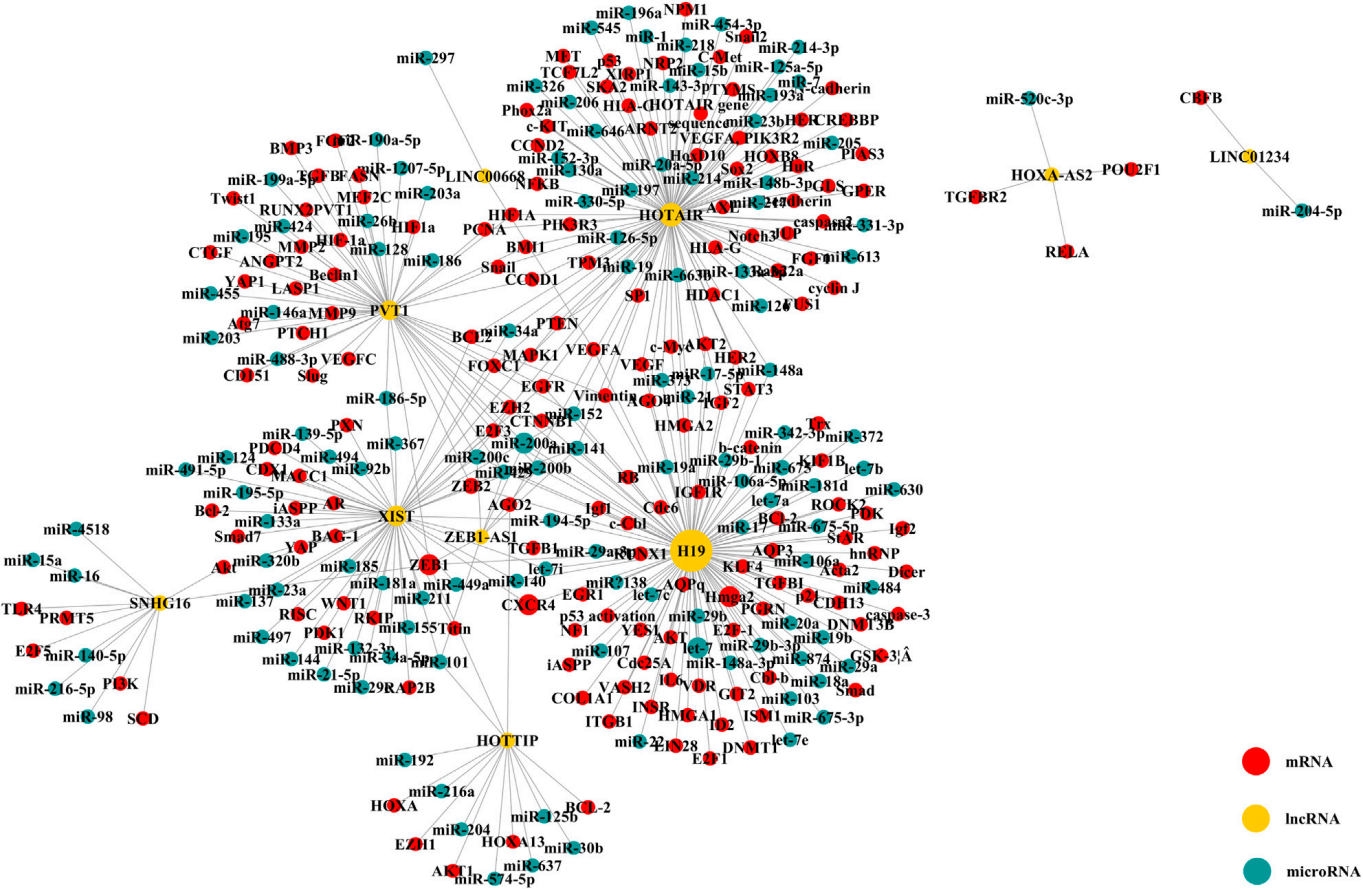
ceRNA network of upregulated lncRNAs (The yellow node means lncRNA; the blue node, microRNA; and the red node, mRNA. The size of node changed with the degree).

Table 4 shows the relationship between dysregulated lncRNAs and proliferation, migration, invasion, and apoptosis of tumor cells, as well as angiogenesis in the included studies. This systematic review and meta-analysis demonstrates the prognostic value of the upregulated lncRNAs on the clinicopathological characteristics and survival of NSCLC. Among the 38 studies of upregulated lncRNAs, 8 reported the mechanism of lncRNAs as prognostic biomarkers in NSCLC through competing with endogenous RNAs (ceRNAs). LncRNA with microRNA response element could be used as a ceRNA to compete with mRNA to bind miRNA, thus, affecting the progression of cancer. Therefore, we searched the lncACTdb database for information of ceRNA of the 32 lncRNAs. To ensure the accuracy of data, we only extracted the experimental terms to construct the ceRNA network, which is shown in Figure 7.

**TABLE 4:** The mechanism of lncRNAs as prognostic biomarkers in NSCLC.

*Cancer* pathogenesis	Upregulated lncRNAs (carcinogens)	Downregulated lncRNAs (antioncogenes)
Proliferation	SNHG18, linc00173.v1, JHDM1D-AS1, MNX1-AS1, BLACAT1, XIST, LBX2-AS1, CACS15, ZEB1-AS1, linc00668, linc00152, H19, PVT1, ANRIL, Sox2ot	GAN1, LOC285194, linc00702, TUSC7
Migration	SNHG18, linc01234, linc00173.v1, MNX1-AS1, BLACAT1, XIST, LBX2-AS1, CACS15, ZEB1-AS1, linc00668, linc00152, ANRIL	LOC285194, linc00702
Invasion	SNHG18, linc01234, JHDM1D-AS1, BLACAT1, XIST, LBX2-AS1, CACS15, ZEB1-AS1, linc00668, linc00152, ANRIL	LOC285194
Apoptosis	MNX1-AS1, linc01234, linc00668, linc00152, PVT1	GAN1, LOC285194, linc00702
Angiogenesis	Linc00173.v1, p21	—

There were 10 lncRNAs, 129 microRNAs, and 173 mRNAs in the ceRNA network. We found that one lncRNA could be involved in several ceRNA networks, which indicated that it can regulate expressions of several mRNAs. Moreover, many mRNAs closely related to the progression and treatment of NSCLC were found to be correlated with lncRNAs, such as ZEB2, p21, hnRNP, EGFR, and EZH2. It was shown that ZEB2 and EZH2 could play a role in cancer metastasis where ZEB2 can be regarded as an epithelial-to-mesenchymal transition (EMT) regulator and EXH2 as a differentiation regulator (Luo et al., 2013; Zhang et al., 2014; Cipollini et al., 2016; Zhang Y et al., 2020; Passiglia et al., 2020; Kashyap et al., 2021; Liu et al., 2021). The CDK-inhibitor p21 (also known as WAF1/SDI1/MDA-6 CIP1/) would result in the arrest of the G1 phase of the cell and effectively suppress tumor growth (Göhring et al., 1999; Vincent et al., 2012). Furthermore, drugs targeting AKT1 may be linked to the phosphorylated p21 (Pérez-Tenorio et al., 2006; Sohn et al., 2006). EGFR is often presented as overexpression in NSCLC patients, and at the same time, EGFR-targeted therapy could improve the survival for patients with positive expression of EGFR (Remon et al., 2018; Meador et al., 2021). Therefore, the dysregulated lncRNAs could be regarded as prognostic biomarkers in NSCLC through regulating the expression of other genes by interacting with other molecules, which further results in transcriptional regulation, epigenetic alteration, sequestration of mRNA or protein, and enhancer regulation.

## Discussion

In this systematic review and meta-analysis, we have tried to include all of the published papers to evaluate the prognostic value of lncRNAs for NSCLC. We found that the studies in the recent 5 years were more normative with lower risk of bias. We have identified that a great deal of dysregulated lncRNAs were associated with prognosis of patients with NSCLC. The results showed that upregulated lncRNAs might imply poor OS in NSCLC (n = 38, HR = 1.79, 95% CI = 1.59–2.02, *p* < 0.01), but downregulated lncRNAs were not significantly associated with OS (n = 10, HR = 0.64, 95% CI:0.3–1.24, *p* = 0.19). In particular, the results indicated that patients with upregulated lncRNAs (linc01234, ZEB1-AS1, linc00152, PVT1, and BANCR) in the TNM Ⅲa stage might be confronted with worse survival (*p* < 0.05).

In this systematic review, 38 upregulated lncRNAs and 10 downregulated lncRNAs in NSCLC were included. The upregulated expression of most lncRNAs was linked to tumor size (n = 16), histological type (n = 18), TNM stage (n = 20), lymph node metastasis (n = 29), differentiation grade (n = 6), and distant metastasis (n = 9), while some were associated with smoking (n = 16), age (n = 20), and gender (n = 33). The high expression of lncRNAs may indicate poor prognosis in NSCLC patients with tumor size larger than 3 cm, LAD type, at the TNM Ⅲ/Ⅳ stage, as well as with positive lymph node metastasis, poor differentiation, or positive distant metastasis. Notably, most of the lncRNAs were related to more than two clinical features of NSCLC. These results confirmed the role of lncRNAs in the pathogenesis and progression of NSCLC, which suggested upregulated lncRNAs indicated poor prognosis. On the contrary, downregulated lncRNAs showed no significant value in terms of clinicopathological features in NSCLC, which may be subject to the amount of publications of downregulated lncRNAs in NSCLC. Even though some downregulated lncRNAs (PINT, linc007002, NBAT1, TCONS00001798, TUSC7, HMlincENA717, and BANCR) were reported to have correlation with lymph node metastasis, TNM stage, and differentiation grade, the meta-analysis indicated no statistical correlation with clinicopathological features of NSCLC. Therefore, if new downregulated lncRNAs were reported in patients with NSCLC, the prognostic value for clinicopathology shall be validated *in vivo* and *in vitro*.

Notably, in the course of clinical treatment of NSCLC, the prognosis of patients at the TNM III stage was diversified, and the clinical decision making was controversial. Our meta-analysis highlighted that the patients with dysregulated lncRNAs (linc01234, ZEB1-AS1, linc00152, PVT1, and BANCR) in the TNM Ⅲa stage may encounter worse prognosis. As for the TNM Ⅲb stage, the lack of valid data made it difficult to analyze, so future studies shall be added in this area. Existing studies demonstrated that dysregulated expression of lncRNAs was significant in multiple cancer tissues and was associated with the TNM stages. A high expression of linc01234 was found to impact the progression of NSCLC and is associated with the TNM stages through functioning as a competing endogenous RNA (*p* < 0.05) (Chen X et al., 2018; Chen Z et al., 2020). Several lncRNAs, including ZEB1-AS1, linc00152, PVT1, and BANCR, were proven to be significantly associated with the advanced TNM stage in liver cancer, bladder cancer, renal cell carcinoma, gastric cancer, and NSCLC, which implied that the combination of dysregulated lncRNAs and TNM stages could contribute to a more accurate determination of prognosis and development of carcinomas (He et al., 2016; Liu et al., 2016; Wang Y et al., 2017; Chen et al., 2017; Zhang et al., 2018; Gao et al., 2019; Jin et al., 2019; Liu et al., 2019; Jiang et al., 2020; Ye et al., 2020; You et al., 2020). Further studies on the differences of lncRNA expression at the TNM Ⅲa, TNM Ⅲb, and TNM Ⅳ stages are needed.

Numerous lncRNAs were aberrantly expressed in NSCLC patients and played critical roles in the progression and metastasis of NSCLC through functioning as either tumor suppressors or oncogenes. In this study, the expression level of lncRNAs was proven to be concerned with NSCLC proliferation (n = 19), invasion (n = 12), migration (n = 14), and apoptosis (n = 8) of tumor cells, which showed that the dysregulation of lncRNAs may impact tumor cell growth and cycle to regulate the prognosis. Resistance to chemotherapy and radiotherapy also poses a great challenge to the treatment of cancer. The inhibition of linc00173 v1 was reported to dramatically enhance sensitivity of LSCC cells to cisplatin, and overexpressed FAM201 A improved the radiosensitivity of NSCLC (Liu et al., 2019; Chen J et al., 2020). Therefore, the dysregulated lncRNAs in NSCLC could imply the clinicopathological changes.

Furthermore, almost all of the published papers (n = 48) reported that lncRNAs were statistically significant predictors for OS of NSCLC. Only a few studies evaluated the associations of lncRNAs expression with other types of survival, including DFS (n = 7), PFS (n = 3), and RFS (n = 1), and there was also a strong connection between them (*p* < 0.05) (Wang Z et al., 2016; Cui et al., 2016; Li and Jiang, 2017; Zhang et al., 2017; Gao et al., 2018; Tian and Feng, 2018; Li et al., 2019; Xu et al., 2019; Zhou et al., 2019; Chen J et al., 2020). The most frequently evaluated lncRNAs in NSCLC included PVT1, ZEB1-AS1, linc01234, H19, AFAP-AS1, and XIST. The expressions of PVT1, ZEB1-AS1, linc01234, and XIST were increased in NSCLC, and the upregulation was associated with a shorter survival. In terms of mechanism, linc01234 could interact with the RNA-binding proteins LSD1 and EZH2, resulting in transcriptional inhibition of BTG2, which affects the development and progression of NSCLC cells through BTG2 epigenetic inhibition (Xie et al., 2019; Chen J et al., 2020). As a competing endogenous RNA, PVT1 could contribute to cell growth and metastasis in NSCLC through regulating miR-361–3p/SOX9 axis and activating the Wnt/β-catenin signaling pathway (Qi and Li, 2020). ZEB1-AS1 was proven to impact cell migration and apoptosis by repressing inhibitor of differentiation-1 (Jin et al., 2019). These results revealed significant prognostic value of lncRNAs for NSCLC survival. Therefore, these lncRNAs may be independent predictors of prognosis in NSCLC.

There was often substantial heterogeneity between studies in the strength of the predictive power. This may be due to the genuine differences of studies, such as different lncRNAs, participants, and different adjustments made in multivariable models. However, it was also highly compatible with the presence of substantial publication bias and other selective reporting bias in this field, which results in exaggerated effects in most small studies and in a high prevalence of nominally significant results. Results of this study failed to reveal a correlation between the dysregulation of lncRNAs in tissues and a poor prognostic of NSCLC patients. For both clinicopathological features and OS, the rate of heterogeneity among studies was very high, which demonstrated that the results of the included studies are strongly conflicting each other (Supplementary Figures S1 and S2). Therefore, we tested the heterogeneity conducting the Begg’s funnel plot, which indicated that publication bias did exist (Supplementary Figures S3 and S4). As for upregulated lncRNAs, we found that several studies (including 2020-Xie-linc00691, 2020-Wang-RAB11B-AS1, 2019-Xu-BLACAT1, 2019-Han-SNHG16, 2019-Liu-FAM201A, 2018-Song-H19, 2017-Li-HOXA-AS2, 2017-Liu-SUMO1P3, 2016-Xue-HOTAIR, and 2015-Deng-AFAP1-AS1) led to a huge risk in bias, which may result from some undiscovered reasons. Despite these heterogeneities, the results were not affected, which is shown from the influential analysis (*p* < 0.0001) (Supplementary Table S5). In 10 deregulated lncRNAs, there were great heterogeneities and influence for OS meta-analysis in NSCLC in three reports (2021-Zhang-Pint, 2020-Wang-NBAT1, and 2014-Xie-HMlinc717). Since the causes of heterogeneity were unidentified, the prognostic value of deregulated lncRNAs for NSCLC was skeptical. Whereas the prognostic studies of lncRNA for NSCLC were increasingly carried out just in recent years, more lncRNAs would come to light and prove to be effective in predicting the clinical process and survival in NSCLC.

Nevertheless, there are some limitations of this review. First, the results of publication bias analysis showed that there were biases in not only upregulated lncRNAs but also in deregulated lncRNAs, which may result from the high publication rate of studies with positive results. On the other hand, the population size from the included studies is not big enough to determine the application of lncRNAs as prognostic biomarkers in clinical practice, considering the tumor stages and tumor size of NSCLC. Thus, data from negative and ongoing studies were underrepresented. Furthermore, the survival of NSCLC patients is subject to treatment, which could influence the estimation of the prognostic value of lncRNAs. Further studies are still warranted.

In conclusion, our meta-analysis suggested that upregulated lncRNAs in NSCLC could serve as the molecular biomarkers to predict the clinicopathological features and prognosis of patients. Specifically, upregulation of five lncRNAs (linc01234, linc00152, PVT1, ZEB1-AS1, and BANCR) combined with the TNM Ⅲa stage, may predict the prognosis of NSCLC more precisely. The prognostic value of deregulated lncRNAs requires further studies. More importantly, lncRNAs can act as ceRNAs or directly bind to miRNAs in the tumorigenesis and growth, which is worth further exploration.

## Data Availability

The original contributions presented in the study are included in the article/Supplementary Material, further inquiries can be directed to the corresponding authors.
