# Supplementary material for: The Value of Dysregulated LncRNAs on Clinicopathology and Survival in Non-Small-Cell Lung Cancer: A Systematic Review and Meta-Analysis
Source: Front Genet. 2022 Apr 5;13:821675. doi: 10.3389/fgene.2022.821675 (PMC9016135; doi:10.3389/fgene.2022.821675)

**Table S1 The detail search terms used in** **Web of Science, Cochrane Library, EBSCO Medline, CBM, China National Knowledge Infrastructure (CNKI), and Wanfang database.**

| Databases | Search stratedies |
| --- | --- |
| PubMed | ((Carcinoma, Non-Small-Cell Lung[MeSH Major Topic]) OR (NSCLC) OR (non small cell lung cancer) OR (Non-Small Cell Lung Cancer) OR (non-small-cell lung cancer) OR (non small cell lung carcinoma) OR (Non-Small-Cell Lung Carcinoma) OR Non-Small-Cell Lung Carcinomas) OR (Adenocarcinoma of Lung[MeSH Major Topic]) OR (Lung Adenocarcinomas) OR (Lung Adenocarcinoma) OR (Adenocarcinoma, Lung) OR (Adenocarcinomas, Lung) OR (lung squamous cell carcinoma) OR (Large cell lung carcinoma) OR (large cell lung cancer) OR (carcinoma,non small cell lung) OR (Carcinoma, Non-Small Cell Lung) OR (carcinomas,non small cell lung) OR (lung carcinoma,non-small-cell) OR (Lung Carcinomas, Non-Small-Cell) OR (Nonsmall Cell Lung Cancer)) AND ((RNA, Long Noncoding[MeSH Major Topic]) OR (Noncoding RNA, Long) OR (lncRNA) OR (Long ncRNA) OR (ncRNA, Long) OR (RNA, Long Non-Translated) OR (Long Non-Translated RNA) OR (Non-Translated RNA, Long) OR (RNA, Long Non Translated) OR (Long Non-Coding RNA) OR (Long Non Coding RNA) OR (Non-Coding RNA, Long) OR (RNA, Long Non-Coding) OR (Long Non-Protein-Coding RNA) OR (Long Non Protein Coding RNA) OR (Non-Protein-Coding RNA, Long) OR (RNA, Long Non-Protein-Coding) OR (Long Noncoding RNA) OR (RNA, Long Untranslated) OR (Long Untranslated RNA) OR (Untranslated RNA, Long) OR (Long ncRNAs) OR (ncRNAs, Long) OR (Long Intergenic Non-Protein Coding RNA) OR (Long Intergenic Non Protein Coding RNA) OR (LincRNAs) OR (LINC RNA)) |
| Web of Sciences | TS=(Carcinoma, Non-Small-Cell Lung OR NSCLC OR non small cell lung cancer OR Non-Small Cell Lung Cancer OR non-small-cell lung cancer OR non small cell lung carcinoma OR Non-Small-Cell Lung Carcinoma OR Non-Small-Cell Lung Carcinomas OR Adenocarcinoma of Lung OR Lung Adenocarcinomas OR Lung Adenocarcinoma OR Adenocarcinoma, Lung OR Adenocarcinomas, Lung OR lung squamous cell carcinoma OR Large cell lung carcinoma OR large cell lung cancer OR carcinoma,non small cell lung OR Carcinoma, Non-Small Cell Lung OR carcinomas,non small cell lung OR lung carcinoma,non-small-cell OR Lung Carcinomas, Non-Small-Cell OR Nonsmall Cell Lung Cancer) AND TS=(RNA, Long Noncoding OR Noncoding RNA, Long OR lncRNA OR Long ncRNA OR ncRNA, Long OR RNA, Long Non-Translated OR Long Non-Translated RNA OR Non-Translated RNA, Long OR RNA, Long Non Translated OR Long Non-Coding RNA OR Long Non Coding RNA OR Non-Coding RNA, Long OR RNA, Long Non-Coding OR Long Non-Protein-Coding RNA OR Long Non Protein Coding RNA OR Non-Protein-Coding RNA, Long OR RNA, Long Non-Protein-Coding OR Long Noncoding RNA OR RNA, Long Untranslated OR Long Untranslated RNA OR Untranslated RNA, Long OR Long ncRNAs OR ncRNAs, Long OR Long Intergenic Non-Protein Coding RNA OR Long Intergenic Non Protein Coding RNA OR LincRNAs OR LINC RNA) |
| Cochrane Library | ((Carcinoma, Non-Small-Cell Lung[MeSH Major Topic]) OR (NSCLC) OR (non small cell lung cancer) OR (Non-Small Cell Lung Cancer) OR (non-small-cell lung cancer) OR (non small cell lung carcinoma) OR (Non-Small-Cell Lung Carcinoma) OR Non-Small-Cell Lung Carcinomas) OR (Adenocarcinoma of Lung[MeSH Major Topic]) OR (Lung Adenocarcinomas) OR (Lung Adenocarcinoma) OR (Adenocarcinoma, Lung) OR (Adenocarcinomas, Lung) OR (lung squamous cell carcinoma) OR (Large cell lung carcinoma) OR (large cell lung cancer) OR (carcinoma,non small cell lung) OR (Carcinoma, Non-Small Cell Lung) OR (carcinomas,non small cell lung) OR (lung carcinoma,non-small-cell) OR (Lung Carcinomas, Non-Small-Cell) OR (Nonsmall Cell Lung Cancer)) AND ((RNA, Long Noncoding[MeSH Major Topic]) OR (Noncoding RNA, Long) OR (lncRNA) OR (Long ncRNA) OR (ncRNA, Long) OR (RNA, Long Non-Translated) OR (Long Non-Translated RNA) OR (Non-Translated RNA, Long) OR (RNA, Long Non Translated) OR (Long Non-Coding RNA) OR (Long Non Coding RNA) OR (Non-Coding RNA, Long) OR (RNA, Long Non-Coding) OR (Long Non-Protein-Coding RNA) OR (Long Non Protein Coding RNA) OR (Non-Protein-Coding RNA, Long) OR (RNA, Long Non-Protein-Coding) OR (Long Noncoding RNA) OR (RNA, Long Untranslated) OR (Long Untranslated RNA) OR (Untranslated RNA, Long) OR (Long ncRNAs) OR (ncRNAs, Long) OR (Long Intergenic Non-Protein Coding RNA) OR (Long Intergenic Non Protein Coding RNA) OR (LincRNAs) OR (LINC RNA)) |
| EBSCO Medline | direct=true&db=mnh&bquery=SU+(+(Carcinoma%2c+Non-Small-Cell+Lung+OR+NSCLC+OR+non+small+cell+lung+cancer+OR+Non-Small+Cell+Lung+Cancer+OR+non-small-cell+lung+cancer+OR+non+small+cell+lung+carcinoma+OR+Non-Small-Cell+Lung+Carcinoma+OR+Non-Small-Cell+Lung+Carcinomas+OR+Adenocarcinoma+of+Lung+OR+Lung+Adenocarcinomas+OR+Lung+Adenocarcinoma+OR+Adenocarcinoma%2c+Lung+OR+Adenocarcinomas%2c+Lung+OR+lung+squamous+cell+carcinoma+OR+carcinoma%2cnon+small+cell+lung+OR+Carcinoma%2c+Non-Small+Cell+Lung+OR+carcinomas%2cnon+small+cell+lung+OR+lung+carcinoma%2cnon-small-cell+OR+Lung+Carcinomas%2c+Non-Small-Cell+OR+Nonsmall+Cell+Lung+Cancer)+)+AND+SU+(+(RNA%2c+Long+Noncoding+OR+Noncoding+RNA%2c+Long+OR+lncRNA+OR+Long+ncRNA+OR+ncRNA%2c+Long+OR+RNA%2c+Long+Non-Translated+OR+Long+Non-Translated+RNA+OR+Non-Translated+RNA%2c+Long+OR+RNA%2c+Long+Non+Translated+OR+Long+Non-Coding+RNA+OR+Long+Non+Coding+RNA+OR+Non-Coding+RNA%2c+Long+OR+RNA%2c+Long+Non-Coding+OR+Long+Non-Protein-Coding+RNA+OR+Long+Non+Protein+Coding+RNA+OR+Non-Protein-Coding+RNA%2c+Long+OR+RNA%2c+Long+Non-Protein-Coding+OR+Long+Noncoding+RNA+OR+RNA%2c+Long+Untranslated+OR+Long+Untranslated+RNA+OR+Untranslated+RNA%2c+Long+OR+Long+ncRNAs+OR+ncRNAs%2c+Long+OR+Long+Intergenic+Non-Protein+Coding+RNA+OR+Long+Intergenic+Non+Protein+Coding+RNA+OR+LincRNAs+OR+LINC+RNA)+)&cli0=DT1&clv0=199501-202012&lang=zh-cn&type=1&searchMode=Standard&site=ehost-live |
| CBM | ((Carcinoma, Non-Small-Cell Lung OR NSCLC OR non small cell lung cancer OR Non-Small Cell Lung Cancer OR non-small-cell lung cancer OR non small cell lung carcinoma OR Non-Small-Cell Lung Carcinoma OR Non-Small-Cell Lung Carcinomas OR Adenocarcinoma of Lung OR Lung Adenocarcinomas OR Lung Adenocarcinoma OR Adenocarcinoma, Lung OR Adenocarcinomas, Lung OR lung squamous cell carcinoma OR Large cell lung carcinoma OR large cell lung cancer OR carcinoma,non small cell lung OR Carcinoma, Non-Small Cell Lung OR carcinomas,non small cell lung OR lung carcinoma,non-small-cell OR Lung Carcinomas, Non-Small-Cell OR Nonsmall Cell Lung Cancer)) AND ((RNA, Long Noncoding OR Noncoding RNA, Long OR lncRNA OR Long ncRNA OR ncRNA, Long OR RNA, Long Non-Translated OR Long Non-Translated RNA OR Non-Translated RNA, Long OR RNA, Long Non Translated OR Long Non-Coding RNA OR Long Non Coding RNA OR Non-Coding RNA, Long OR RNA, Long Non-Coding OR Long Non-Protein-Coding RNA OR Long Non Protein Coding RNA OR Non-Protein-Coding RNA, Long OR RNA, Long Non-Protein-Coding OR Long Noncoding RNA OR RNA, Long Untranslated OR Long Untranslated RNA OR Untranslated RNA, Long OR Long ncRNAs OR ncRNAs, Long OR Long Intergenic Non-Protein Coding RNA OR Long Intergenic Non Protein Coding RNA OR LincRNAs OR LINC RNA)) |
| China National Knowledge Infrastructure (CNKI) | SU = '非小细胞肺癌'+‘NSCLC’+‘肺腺癌’+‘肺鳞癌’+‘肺鳞状细胞癌’ AND SU = '长链非编码rna'+‘长的非编码RNA’+‘长非编码RAN’+‘lnc RNA’ |
| Wanfang | (全部:(“非小细胞肺癌”or“NSCLC”or“肺腺癌”or“肺鳞癌”or“肺鳞状细胞癌”or“肺大细胞癌”or“大细胞肺癌”）and（“lncRNA”or“长链非编码RNA”or“长的非编码RNA”or“长非编码RNA”）)*Date:1995- |

**Table S2 Summary of the Bias Domains, Prompting Items, and Ratings of the QUIPS Tool**

| Variable | Bias Domains | | | | | |
| --- | --- | --- | --- | --- | --- | --- |
|  | 1. Study Participation | 2. Study Attrition | 3. Prognostic Factor Measurement | 4. Outcome Measurement | 5. Study Confounding | 6. Statistical Analysis and Reporting |
| Optimal study or characteristics of unbiased study | The study sample adequately represents the population of interest | The study data available (i.e.,participants not lost to follow-up) adequately represent the study sample | The PF is measured in a similar way for all participants | The outcome of interest is measured in a similar  way for all participants | Important potential confounding factors are appropriately accounted for | The statistical analysis is appropriate, and all primary outcomes are reported |
| Prompting items and  considerations† | a. Adequate participation in  the study by eligible persons | a. Adequate response rate for study participants | a. A clear definition or  description of the PF is provided | a. A clear definition of the  outcome is provided | a. All important confounders are measured | a. Sufficient presentation of data to assess the adequacy of the analytic strategy |
|  | b. Description of the source  population or population  of interest | b. Description of attempts to collect information on participants who dropped out | b. Method of PF measurement is adequately valid and reliable | b. Method of outcome measurement used is adequately valid and reliable | b. Clear definitions of the important confounders measured are provided | b. Strategy for model building is appropriate and is based on a conceptual framework or model |
|  | c. Description of the baseline  study sample | c. Reasons for loss to follow-up are  provided | c. Continuous variables are reported or appropriate cut points are used | c. The method and setting of outcome measurement is the  same for all study participants | c. Measurement of all important confounders is adequately valid and reliable | c. The selected statistical model is adequate for the design of the study |
|  | d. Adequate description of the sampling frame and recruitment | d. Adequate description of  participants lost to follow-up | d. The method and setting of measurement of PF is the same or all study participants |  | d. The method and setting of confounding measurement are the same for all study participants | d. There is no selective reporting of results |
|  | e. Adequate description of the period and place of recruitment | e. There are no important differences between participants who completed the study and those who did not | e. Adequate proportion of the study sample has complete data for the PF |  | e. Appropriate methods are used if imputation is used for missing confounder data |  |
|  | f. Adequate description of inclusion and exclusion criteria |  | f. Appropriate methods of imputation are used for missing PF data |  | f. Important potential confounders are accounted for in the study design |  |
|  |  |  |  |  | g. Important potential confounders are accounted for in the analysis |  |
| Ratings‡ | | | | | | |
| High risk of bias | The relationship between the PF and outcome is very likely to be different for  participants and eligible nonparticipants | The relationship between the PF and outcome is very likely to be different for completing and noncompleting participants | The measurement of the PF is very likely to be different for different levels of the outcome of interest | The measurement of the outcome is very likely to be different related to the baseline level of the PF | The observed effect of the PF on the outcome is very likely to be distorted by another factor related to PF and outcome | The reported results are very likely to be spurious or biased related to analysis or reporting |
| Moderate risk of bias | The relationship between the PF and outcome may be different for participants  and eligible nonparticipants | The relationship between the PF and outcome may be different for  completing and noncompleting participants | The measurement of the PF may be different for different levels of the outcome of interest | The measurement of the outcome may be different related to the baseline level of the PF | The observed effect of the PF on outcome may be distorted by another factor related to PF and outcome | The reported results may be spurious or biased related to analysis or reporting |
| Low risk of bias | The relationship between the PF and outcome is unlikely to be different for  participants and eligible nonparticipants | The relationship between the PF and outcome is unlikely to be different for completing and noncompleting participants | The measurement of the PF is unlikely to be different for  different levels of the outcome of interest | The measurement of the outcome is unlikely to be different related to the baseline level of the PF | The observed effect of the PF on outcome is unlikely to be distorted by another factor related to PF and outcome | The reported results are unlikely to be spurious or biased related to analysis or reporting |

**Table S3 The quality assessment for 48 studies in the meta-analysis**

| Studies | Study Participation | | | | | | Study Attrition | | | | | Prognostic Factor Measurement | | | | | | Outcome Measurement | | | Study Confounding | | | | | | | Statistical Analysis and Reporting | | | | score | level |
| --- | --- | --- | --- | --- | --- | --- | --- | --- | --- | --- | --- | --- | --- | --- | --- | --- | --- | --- | --- | --- | --- | --- | --- | --- | --- | --- | --- | --- | --- | --- | --- | --- | --- |
|  | a | b | c | d | e | f | a | b | c | d | e | a | b | c | d | e | f | a | b | c | a | b | c | d | e | f | g | a | b | c | d |  |  |
| 2021,Wang,FAM83A-AS1 | √ | √ | √ | √ | √ | √ | √ | √ | √ | √ | √ | √ | √ | √ | √ | √ | √ |  | √ | √ |  | √ | √ | √ |  | √ | √ | √ | √ | √ | √ | 28 | high |
| 2021,Fan,SNHG18 | √ |  | √ |  |  |  | √ | √ | √ | √ | √ | √ | √ | √ | √ | √ | √ |  | √ | √ |  |  |  |  |  |  |  | √ | √ | √ | √ | 19 | low |
| 2020,Xie,linc00691 | √ | √ | √ |  | √ | √ | √ | √ | √ | √ | √ | √ | √ | √ | √ | √ | √ |  | √ | √ | √ | √ | √ | √ |  | √ | √ | √ | √ | √ | √ | 28 | high |
| 2020,Wang,TDRG1 | √ | √ | √ |  | √ |  | √ | √ | √ | √ | √ | √ | √ | √ | √ | √ | √ |  | √ | √ |  | √ | √ | √ |  | √ | √ | √ | √ | √ | √ | 26 | high |
| 2020,Wang,RAB11B-AS1 | √ | √ | √ |  | √ | √ | √ | √ | √ | √ | √ | √ | √ | √ | √ | √ | √ |  | √ | √ |  |  | √ | √ |  | √ | √ | √ | √ | √ | √ | 26 | high |
| 2020,Ma,linc00504 | √ |  |  |  | √ | √ | √ | √ | √ | √ | √ | √ | √ | √ | √ | √ | √ |  | √ | √ |  |  | √ | √ |  | √ | √ | √ | √ | √ | √ | 24 | moderate |
| 2020,Hua,lncRNA-AC020978 | √ | √ |  |  | √ |  | √ | √ | √ | √ | √ | √ | √ | √ | √ | √ | √ |  | √ | √ |  |  | √ | √ |  | √ | √ | √ | √ | √ | √ | 24 | moderate |
| 2020,Fang,XIST | √ | √ | √ |  | √ |  | √ | √ | √ | √ | √ | √ | √ |  | √ | √ | √ |  | √ | √ |  | √ | √ | √ |  | √ | √ | √ | √ | √ | √ | 25 | moderate |
| 2020,Chen,linc01234 | √ | √ |  | √ | √ |  | √ | √ | √ | √ | √ | √ | √ | √ | √ | √ | √ |  | √ | √ |  |  | √ | √ |  | √ | √ | √ | √ | √ | √ | 25 | moderate |
| 2020,Chen,linc00173.v1 | √ | √ | √ | √ | √ |  | √ | √ | √ | √ | √ | √ | √ | √ | √ | √ | √ |  | √ | √ |  |  | √ | √ |  | √ | √ | √ | √ | √ | √ | 26 | high |
| 2020,Castellano,p21 | √ | √ | √ |  |  |  | √ | √ | √ | √ | √ | √ | √ | √ | √ | √ | √ |  | √ | √ |  | √ | √ | √ |  | √ | √ | √ | √ | √ | √ | 25 | moderate |
| 2019,Yao,JHDM1D-AS1 | √ |  |  |  | √ |  | √ | √ | √ | √ | √ | √ | √ |  | √ | √ | √ |  | √ | √ |  |  | √ | √ |  | √ | √ | √ | √ | √ | √ | 22 | moderate |
| 2019,Yang,MNX1-AS1 | √ |  |  |  |  |  | √ | √ | √ | √ | √ | √ | √ |  | √ | √ | √ |  | √ | √ |  |  | √ | √ |  | √ | √ | √ | √ | √ | √ | 21 | moderate |
| 2019,Xu,BLACAT1 | √ | √ | √ | √ | √ | √ | √ | √ | √ | √ | √ | √ | √ | √ | √ | √ | √ | √ | √ | √ |  |  | √ | √ |  |  |  | √ | √ | √ | √ | 26 | high |
| 2019,Xie,linc01234 | √ |  |  |  | √ |  | √ | √ | √ | √ | √ | √ | √ | √ | √ | √ | √ |  | √ | √ |  |  | √ | √ |  | √ | √ | √ | √ | √ | √ | 23 | moderate |
| 2019,Wang,XIST | √ |  |  |  | √ |  | √ | √ | √ | √ | √ | √ | √ | √ | √ | √ | √ |  | √ | √ | √ | √ | √ | √ |  | √ | √ | √ | √ | √ | √ | 25 | moderate |
| 2019,Tang,LBX2-AS1 | √ |  | √ |  |  |  | √ | √ | √ | √ | √ | √ | √ | √ | √ | √ | √ |  | √ | √ |  |  | √ | √ |  | √ | √ | √ | √ | √ | √ | 23 | moderate |
| 2019,Navarro,HOTTIP | √ | √ | √ |  | √ |  | √ | √ | √ | √ | √ | √ | √ | √ | √ | √ | √ | √ | √ | √ |  | √ | √ | √ |  | √ | √ | √ | √ | √ | √ | 27 | high |
| 2019,Liu,FAM201A | √ |  |  |  | √ |  | √ | √ | √ | √ | √ | √ | √ | √ | √ | √ | √ |  | √ | √ |  |  | √ | √ |  | √ | √ | √ | √ | √ | √ | 23 | moderate |
| 2019,Li,CACS15 | √ |  |  |  | √ |  | √ | √ | √ | √ | √ | √ | √ | √ | √ | √ | √ |  | √ | √ |  |  | √ | √ |  | √ | √ | √ | √ | √ | √ | 23 | moderate |
| 2019,Jin,ZEB1-AS1 | √ | √ | √ |  | √ |  | √ | √ | √ | √ | √ | √ | √ | √ | √ | √ | √ |  | √ | √ |  |  | √ | √ |  | √ | √ | √ |  |  | √ | 23 | moderate |
| 2019,Han,SNHG16 | √ |  |  |  | √ |  | √ | √ | √ | √ | √ | √ | √ | √ | √ | √ | √ | √ | √ | √ |  |  | √ | √ |  | √ | √ | √ | √ | √ | √ | 24 | moderate |
| 2019,An,linc00668 | √ | √ |  | √ | √ | √ | √ | √ | √ | √ | √ | √ | √ | √ | √ | √ | √ |  | √ | √ |  |  | √ | √ |  | √ | √ | √ | √ | √ | √ | 26 | high |
| 2018,Yin,AFAP1-AS1 | √ | √ |  |  |  |  | √ | √ | √ | √ | √ | √ | √ | √ | √ | √ | √ |  | √ | √ |  |  | √ | √ |  | √ | √ | √ | √ | √ | √ | 23 | moderate |
| 2018,Xie,ZEB1-AS1 | √ | √ |  |  |  |  | √ | √ | √ | √ | √ | √ | √ | √ | √ | √ | √ |  | √ | √ |  |  | √ | √ |  | √ | √ | √ | √ | √ | √ | 23 | moderate |
| 2018,Tian,uc.338 | √ | √ | √ |  | √ |  | √ | √ | √ | √ | √ | √ | √ | √ | √ | √ | √ |  | √ | √ |  |  | √ | √ |  | √ | √ | √ | √ | √ | √ | 25 | moderate |
| 2018,Song,H19 | √ | √ | √ |  | √ | √ | √ | √ | √ | √ | √ | √ | √ | √ | √ | √ | √ |  | √ | √ |  |  | √ | √ |  | √ | √ | √ | √ | √ | √ | 26 | high |
| 2017,Zhang,linc00152 | √ | √ |  |  | √ |  | √ | √ | √ | √ | √ | √ | √ | √ | √ | √ | √ | √ | √ | √ |  | √ | √ | √ |  | √ | √ | √ | √ | √ | √ | 26 | high |
| 2017,Liu,SUMO1P3 | √ |  |  |  | √ |  | √ | √ | √ | √ | √ | √ | √ | √ | √ | √ | √ | √ | √ | √ |  |  | √ | √ |  | √ | √ | √ | √ | √ | √ | 24 | moderate |
| 2017,Li,HOXA-AS2 | √ | √ |  |  |  |  | √ | √ | √ | √ | √ | √ | √ | √ | √ | √ | √ |  | √ | √ |  | √ | √ | √ |  | √ | √ | √ |  | √ | √ | 23 | moderate |
| 2016,Zhang,H19 | √ | √ | √ |  |  |  | √ | √ | √ | √ | √ | √ | √ | √ | √ | √ | √ | √ | √ | √ |  | √ | √ | √ |  | √ | √ | √ | √ | √ | √ | 26 | high |
| 2016,Xue,HOTAIR | √ | √ | √ |  | √ |  | √ | √ | √ | √ | √ | √ | √ | √ | √ | √ | √ | √ | √ | √ |  |  | √ | √ |  | √ | √ | √ | √ | √ | √ | 26 | high |
| 2016,Wan,PVT1 | √ | √ | √ |  | √ |  | √ | √ | √ | √ | √ | √ | √ | √ | √ | √ | √ | √ | √ | √ |  |  | √ | √ |  | √ | √ | √ | √ | √ | √ | 26 | high |
| 2016,Cui,PVT1 | √ | √ |  |  | √ |  | √ | √ | √ | √ | √ | √ | √ | √ | √ | √ | √ | √ | √ | √ |  |  | √ | √ |  | √ | √ | √ | √ | √ | √ | 25 | moderate |
| 2015,Wang,UCA1 | √ |  |  |  | √ |  | √ | √ | √ | √ | √ | √ | √ |  | √ | √ | √ |  | √ | √ |  |  |  | √ |  |  |  | √ | √ | √ | √ | 19 | low |
| 2015,Lin,ANRIL | √ | √ |  |  | √ |  | √ | √ | √ | √ | √ | √ | √ | √ | √ | √ | √ |  | √ | √ |  |  | √ | √ |  | √ | √ | √ | √ | √ | √ | 24 | moderate |
| 2015,Deng,AFAP1-AS1 | √ | √ | √ |  | √ |  | √ | √ | √ | √ | √ | √ | √ | √ | √ | √ | √ | √ | √ | √ |  |  | √ | √ |  | √ | √ | √ |  |  | √ | 24 | moderate |
| 2014,Hou,Sox2ot | √ | √ |  |  | √ |  | √ | √ | √ | √ | √ | √ | √ | √ | √ | √ | √ | √ | √ | √ |  |  | √ | √ |  | √ | √ | √ | √ | √ | √ | 25 | moderate |
| 2021,Zhang,PINT | √ | √ | √ | √ | √ | √ | √ | √ | √ | √ | √ | √ | √ | √ | √ | √ | √ |  | √ | √ |  | √ | √ | √ |  | √ | √ | √ | √ | √ | √ | 28 | high |
| 2021,Wang,GAN1 | √ |  | √ |  | √ |  | √ | √ | √ | √ | √ | √ | √ | √ | √ | √ | √ |  | √ | √ |  | √ | √ | √ |  | √ | √ | √ | √ | √ | √ | 25 | moderate |
| 2020,Wang,NBAT1 | √ | √ | √ |  | √ | √ | √ | √ | √ | √ | √ | √ | √ | √ | √ | √ | √ | √ | √ | √ |  | √ | √ | √ |  | √ | √ | √ | √ | √ | √ | 28 | high |
| 2019,Zhou,LOC285194 | √ | √ | √ |  | √ |  | √ | √ | √ | √ | √ | √ | √ | √ | √ | √ | √ |  | √ | √ |  | √ | √ | √ |  | √ | √ | √ | √ | √ | √ | 26 | high |
| 2019,Yu,linc00702 | √ | √ | √ |  | √ |  | √ | √ | √ | √ | √ | √ | √ |  | √ | √ | √ |  | √ | √ |  |  | √ | √ |  | √ | √ | √ |  |  | √ | 22 | moderate |
| 2018,Gao,TCONS00001798 | √ | √ | √ |  | √ |  | √ | √ | √ | √ | √ | √ | √ | √ | √ | √ | √ | √ | √ | √ |  | √ | √ | √ |  | √ | √ | √ | √ | √ | √ | 27 | high |
| 2016,Wang,TUSC7 | √ | √ |  |  | √ |  | √ | √ | √ | √ | √ | √ | √ | √ | √ | √ | √ | √ | √ | √ |  |  | √ | √ |  | √ | √ | √ | √ | √ | √ | 25 | moderate |
| 2014,Xie,HMlincRNA717 | √ | √ |  |  | √ |  | √ | √ | √ | √ | √ | √ | √ | √ | √ | √ | √ |  | √ | √ |  |  | √ | √ |  | √ | √ | √ | √ | √ | √ | 24 | moderate |
| 2014,Sun,BANCR | √ | √ |  |  | √ |  | √ | √ | √ | √ | √ | √ | √ | √ | √ | √ | √ |  | √ | √ |  |  | √ | √ |  | √ | √ | √ | √ | √ | √ | 24 | moderate |
| 2013,Han,GAS6-AS1 | √ | √ |  |  | √ |  | √ | √ | √ | √ | √ | √ | √ |  | √ | √ | √ |  | √ | √ |  |  | √ | √ |  |  |  | √ | √ | √ | √ | 21 | moderate |

**Table S4 The clinical characteristics of patients with dysregulated lncRNAs in NSCLC.**

|  | studies | samples | | P | I^2^ | model | HR | 95%CI |
| --- | --- | --- | --- | --- | --- | --- | --- | --- |
|  |  | experimental | control |  |  |  |  |  |
| upregulated lncRNAs clinicopathology | | | | | | | | |
| Age |  |  |  |  |  |  |  |  |
| ≤60 | 20 | 1243 | 1116 | 0.97 | 20% | fixed | 1.00 | [0.85, 1.19] |
| ＞60 | 20 | 1243 | 1116 | 0.97 | 20% | fixed | 1.00 | [0.84, 1.18] |
| gender |  |  |  |  |  |  |  |  |
| male | 33 | 1943 | 1761 | 0.58 | 2% | fixed | 1.04 | [0.91, 1.19] |
| female | 33 | 1943 | 1780 | 0.58 | 2% | fixed | 0.96 | [0.84, 1.10] |
| Tumor size |  |  |  |  |  |  |  |  |
| ≤3 cm | 16 | 862 | 832 | ＜0.01 | 52% | random | 0.52 | [0.43, 0.64] |
| ＞3 cm | 16 | 862 | 832 | ＜0.01 | 52% | random | 1.92 | [1.57, 2.34] |
| Smoke |  |  |  |  |  |  |  |  |
| Never | 16 | 906 | 862 | 0.94 | 28% | fixed | 0.99 | [0.82, 1.21] |
| Ever | 16 | 894 | 862 | 0.72 | 44% | fixed | 1.04 | [0.85, 1.26] |
| Histological type |  |  |  |  |  |  |  |  |
| LSCC | 18 | 953 | 873 | ＜0.01 | 0% | fixed | 0.78 | [0.65, 0.95] |
| LAD | 18 | 953 | 873 | ＜0.01 | 11% | fixed | 1.30 | [1.08, 1.57] |
| TNM stages |  |  |  |  |  |  |  |  |
| Ⅰ/Ⅱ | 20 | 1137 | 1094 | ＜0.01 | 70% | random | 0.41 | [0.29, 0.57] |
| Ⅲ/Ⅳ | 20 | 1137 | 1094 | ＜0.01 | 70% | random | 2.44 | [1.73, 3.44] |
| Lymph node metastasis |  |  |  |  |  |  |  |  |
| Negative | 29 | 1555 | 1445 | ＜0.01 | 81% | random | 0.49 | [0.34, 0.71] |
| Positive | 29 | 1555 | 1445 | ＜0.01 | 81% | random | 2.04 | [1.40, 2.96] |
| Differentiation |  |  |  |  |  |  |  |  |
| Well/moderate | 6 | 355 | 332 | 0.04 | 56% | random | 0.61 | [0.38, 0.99] |
| Poor | 6 | 355 | 332 | 0.04 | 56% | random | 1.63 | [1.01, 2.64] |
| Distant metastasis |  |  |  |  |  |  |  |  |
| Negative | 9 | 480 | 446 | ＜0.01 | 43% | fixed | 0.37 | [0.26, 0.53] |
| Positive | 9 | 480 | 446 | ＜0.01 | 43% | fixed | 2.72 | [1.90, 3.90] |
| downregulated lncRNAs clinicopathology | | | | | | | | |
| Age |  |  |  |  |  |  |  |  |
| ≤60 | 6 | 280 | 296 | 0.49 | 0% | fixed | 1.08 | [0.77,1.52] |
| ＞60 | 6 | 284 | 287 | 0.49 | 0% | fixed | 0.93 | [0.66,1.30] |
| gender |  |  |  |  |  |  |  |  |
| male | 8 | 413 | 438 | 0.7 | 0% | fixed | 1.04 | [0.79,1.37] |
| female | 8 | 413 | 438 | 0.7 | 0% | fixed | 0.96 | [0.73;1.27] |
| Tumor size |  |  |  |  |  |  |  |  |
| ≤3 cm | 5 | 253 | 267 | 0.87 | 70% | random | 1.06 | [0.55,2.05] |
| ＞3 cm | 5 | 253 | 267 | 0.87 | 70% | random | 0.94 | [0.49,1.83] |
| Smoke |  |  |  |  |  |  |  |  |
| Never | 6 | 308 | 313 | 0.55 | 0% | fixed | 1.10 | [0.80,1.53] |
| Ever | 6 | 308 | 313 | 0.55 | 0% | fixed | 0.91 | [0.65,1.25] |
| Histological type |  |  |  |  |  |  |  |  |
| LSCC | 7 | 336 | 368 | 0.55 | 48% | fixed | 1.10 | [0.81,1.50] |
| LAD | 7 | 336 | 368 | 0.55 | 48% | fixed | 0.91 | [0.67,1.24] |
| TNM stages |  |  |  |  |  |  |  |  |
| Ⅰ/Ⅱ | 3 | 147 | 143 | 0.85 | 89% | random | 1.15 | [0.27,4.96] |
| Ⅲ/Ⅳ | 3 | 147 | 143 | 0.85 | 89% | random | 0.87 | [0.20,3.74] |
| Lymph node metastasis |  |  |  |  |  |  |  |  |
| Negative | 6 | 303 | 329 | 0.84 | 85% | random | 1.09 | [0.46,2.58] |
| Positive | 6 | 303 | 329 | 0.84 | 85% | random | 0.91 | [0.39,2.16] |

**Table S5 The influential analysis for overall survival of upregulated lncRNAs in non-small-cell lung cancer.**

| studies | HR | 95%-CI | p-value | tau^2 | tau |
| --- | --- | --- | --- | --- | --- |
| 2021,Wang,FAM83A-AS1 | 1.7763 | [1.5774,2.0002] | <0.0001 | 0.05 | 0.2313 |
| 2021,Fan,SNHG18 | 1.7664 | [1.5690,1.9887] | <0.0001 | 0.05 | 0.2298 |
| 2020,Xie,linc00691 | 1.7555 | [1.5601,1.9753] | <0.0001 | 0.05 | 0.228 |
| 2020,Ma,linc00504 | 1.7613 | [1.5650,1.9822] | <0.0001 | 0.05 | 0.2292 |
| 2020,Wang,RAB11B-AS1 | 1.7482 | [1.5537,1.9671] | <0.0001 | 0.05 | 0.2247 |
| 2020,Hua,lncRNA-AC020978 | 1.7727 | [1.5746,1.9958] | <0.0001 | 0.05 | 0.2309 |
| 2020,Chen,linc01234 | 1.7732 | [1.5759,1.9953] | <0.0001 | 0.05 | 0.2314 |
| 2020,Chen,linc00173.v1 | 1.7886 | [1.5871,2.0157] | <0.0001 | 0.05 | 0.232 |
| 2020,Castellano,p21 | 1.7716 | [1.5748,1.9930] | <0.0001 | 0.05 | 0.2311 |
| 2020,Wang,TDRG1 | 1.8198 | [1.6173,2.0476] | <0.0001 | 0.05 | 0.2307 |
| 2020,Fang,XIST | 1.7692 | [1.5719,1.9913] | <0.0001 | 0.05 | 0.2306 |
| 2019,Yao,JHDM1D-AS1 | 1.7707 | [1.5721,1.9944] | <0.0001 | 0.05 | 0.2298 |
| 2019,Yang,MNX1-AS1 | 1.7882 | [1.5869,2.0150] | <0.0001 | 0.05 | 0.2321 |
| 2019,Xu,BLACAT1 | 1.7591 | [1.5631,1.9797] | <0.0001 | 0.05 | 0.2214 |
| 2019,Xie,linc01234 | 1.7622 | [1.5659,1.9830] | <0.0001 | 0.05 | 0.2295 |
| 2019,Wang,XIST | 1.7794 | [1.5799,2.0042] | <0.0001 | 0.05 | 0.2315 |
| 2019,Tang,LBX2-AS1 | 1.7615 | [1.5653,1.9822] | <0.0001 | 0.05 | 0.2293 |
| 2019,Li,CACS15 | 1.8368 | [1.6297,2.0702] | <0.0001 | 0.05 | 0.2324 |
| 2019,Han,SNHG16 | 1.8374 | [1.6350,2.0648] | <0.0001 | 0.05 | 0.2258 |
| 2019,An,linc00668 | 1.7603 | [1.5642,1.9809] | <0.0001 | 0.05 | 0.2291 |
| 2019,Liu,FAM201A | 1.8645 | [1.6558,2.0994] | <0.0001 | 0.05 | 0.2285 |
| 2019,Navarro,HOTTIP | 1.7751 | [1.5770,1.9981] | <0.0001 | 0.05 | 0.2314 |
| 2019,Jin,ZEB1-AS1 | 1.7761 | [1.5775,1.9997] | <0.0001 | 0.05 | 0.2314 |
| 2018,Xie,ZEB1-AS1 | 1.792 | [1.5896,2.0202] | <0.0001 | 0.05 | 0.232 |
| 2018,Tian,uc.338 | 1.7637 | [1.5673,1.9848] | <0.0001 | 0.05 | 0.2298 |
| 2018,Yin,AFAP1-AS1 | 1.9062 | [1.5577,2.3327] | <0.0001 | 0.28 | 0.5331 |
| 2018,Song,H19 | 1.7694 | [1.5706,1.9933] | <0.0001 | 0.05 | 0.2268 |
| 2017,Zhang,linc00152 | 1.7691 | [1.5722,1.9907] | <0.0001 | 0.05 | 0.2308 |
| 2017,Li,HOXA-AS2 | 1.7386 | [1.5474,1.9535] | <0.0001 | 0.05 | 0.2244 |
| 2017,Liu,SUMO1P3 | 1.8257 | [1.6237,2.0528] | <0.0001 | 0.05 | 0.2283 |
| 2016,Cui,PVT1 | 1.7829 | [1.5825,2.0086] | <0.0001 | 0.05 | 0.2316 |
| 2016,Xue,HOTAIR | 1.7539 | [1.5584,1.9739] | <0.0001 | 0.05 | 0.2271 |
| 2016,Wan,PVT1 | 1.7709 | [1.5732,1.9934] | <0.0001 | 0.05 | 0.2308 |
| 2016,Zhang,H19 | 1.9046 | [1.5469,2.3449] | <0.0001 | 0.31 | 0.5545 |
| 2015,Wang,UCA1 | 1.7787 | [1.5792,2.0033] | <0.0001 | 0.05 | 0.2314 |
| 2015,Lin,ANRIL | 1.7691 | [1.5717,1.9914] | <0.0001 | 0.05 | 0.2305 |
| 2015,Deng,AFAP1-AS1 | 1.7496 | [1.5568,1.9662] | <0.0001 | 0.05 | 0.2265 |
| 2014,Hou,Sox2ot | 1.7716 | [1.5743,1.9936] | <0.0001 | 0.05 | 0.2311 |
| Pooled estimate | 1.7833 | [1.5855,2.0058] | <0.0001 | 0.05 | 0.2322 |

|  | HR | 95%-CI | p-value | tau^2 | tau |
| --- | --- | --- | --- | --- | --- |
| 2021,Wang,GAN1 | 0.6445 | [0.3000;1.3843] | 0.2601 | 1.1773 | 1.085 |
| 2021,Zhang,PINT | 0.5473 | [0.2892;1.0357] | 0.064 | 0.7724 | 0.8789 |
| 2020,Wang,NBAT1 | 0.5426 | [0.2815;1.0459] | 0.0678 | 0.8326 | 0.9125 |
| 2019,Zhou,LOC285194 | 0.7423 | [0.3842;1.4344] | 0.3753 | 0.8444 | 0.9189 |
| 2019,Yu,linc00702 | 0.698 | [0.3526;1.3815] | 0.302 | 0.9374 | 0.9682 |
| 2018,Gao,TCONS00001798 | 0.674 | [0.3268;1.3900] | 0.2854 | 1.0405 | 1.0201 |
| 2016,Wang,TUSC7 | 0.71 | [0.3590;1.4044] | 0.3251 | 0.9245 | 0.9615 |
| 2014,Xie,HMlincRNA717 | 0.5509 | [0.2812;1.0790] | 0.0822 | 0.88 | 0.9381 |
| 2014,Sun,BANCR | 0.6604 | [0.3212;1.3582] | 0.2594 | 1.0362 | 1.018 |
| 2013,Han,GAS6-AS1 | 0.7122 | [0.3655;1.3879] | 0.3188 | 0.9143 | 0.9562 |
| Pooled estimate | 0.6449 | [0.3366;1.2358] | 0.1862 | 0.9294 | 0.9641 |
| After delete three studies with high heterogeneity (2021-Zhang-PINT, 2020-Wang-NBAT1, and 2014-Xie-HMlincRNA717): | | | | | |
|  | HR | 95%-CI | p-value | tau^2 | tau |
| 2021,Wang,GAN1 | 0.3445 | [0.2528;0.4696] | <0.0001 | 0.0103 | 0.1016 |
| 2019,Zhou,LOC285194 | 0.4503 | [0.3457;0.5867] | <0.0001 | 0 | 0 |
| 2019,Yu,linc00702 | 0.4196 | [0.3250;0.5417] | <0.0001 | 0.0781 | 0.2795 |
| 2018,Gao,TCONS00001798 | 0.4076 | [0.3056;0.5436] | <0.0001 | 0.1194 | 0.3456 |
| 2016,Wang,TUSC7 | 0.4264 | [0.3292;0.5524] | <0.0001 | 0.0685 | 0.2617 |
| 2014,Sun,BANCR | 0.398 | [0.3035;0.5219] | <0.0001 | 0.0947 | 0.3077 |
| 2013,Han,GAS6-AS1 | 0.4202 | [0.3266;0.5407] | <0.0001 | 0.0571 | 0.239 |
| Pooled estimate | 0.4116 | [0.3207;0.5283] | <0.0001 | 0.0614 | 0.2477 |

**Table S6 The influential analysis for overall survival of downregulated lncRNAs in non-small-cell lung cancer.**

**Figure S1 the heterogeneity analysis for overall survival of upregulalted lncRNAs in non-small-cell lung cancer.**


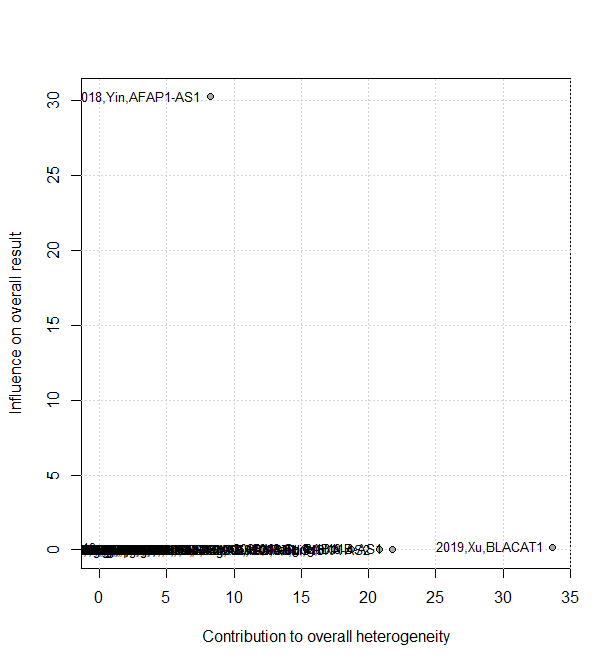


**Figure S2 The heterogeneity analysis for overall survival of downregulalted lncRNAs in non-small-cell lung cancer.**


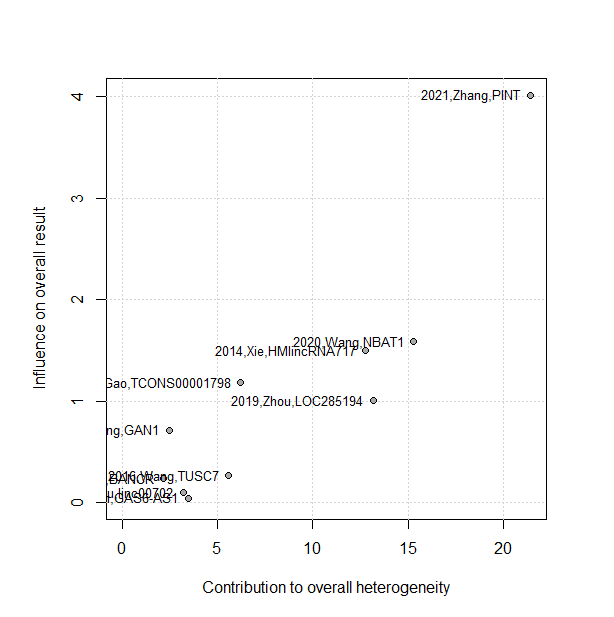


**Figure S3 Begg’s funnel plot for publication bias on the association between the upregulation of lncRNAs and non-small cell lung cancer overall survial.**


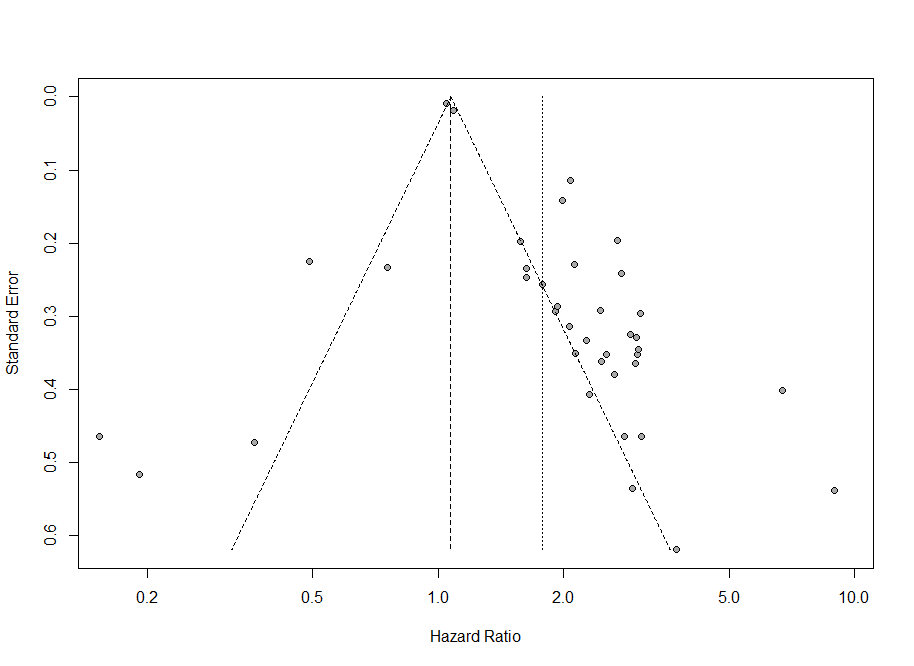


**Figure S4 Begg’s funnel plot for publication bias on the association between the downewgulation of lncRNAs and non-small cell lung cancer overall survial.**


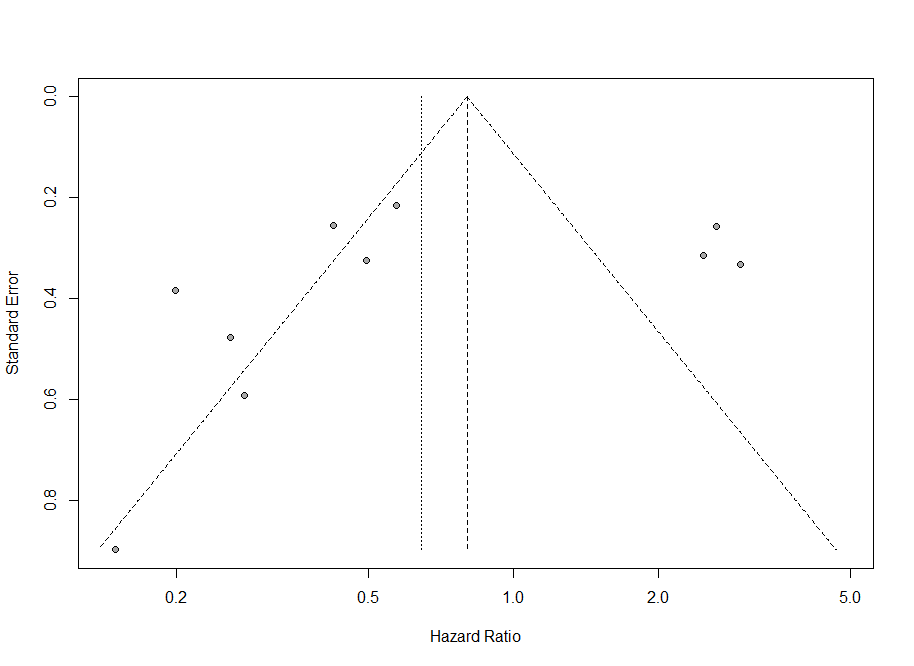

Supplement: Supplementary file 1 [file DataSheet1.docx]
